# The Transcription Factor ZafA Regulates the Homeostatic and Adaptive Response to Zinc Starvation in *Aspergillus fumigatus*

**DOI:** 10.3390/genes9070318

**Published:** 2018-06-26

**Authors:** Rocío Vicentefranqueira, Jorge Amich, Laura Marín, Clara Inés Sánchez, Fernando Leal, José Antonio Calera

**Affiliations:** 1 Instituto de Biología Funcional y Genómica (IBFG-CSIC), Departamento de Microbiología y Genética, Universidad de Salamanca, 37007 Salamanca, Spain; rvrdew@usal.es (R.V.); lauramarin@usal.es (L.M.); cisanchezs@usal.es (C.I.S.); fleal@usal.es (F.L.); 2 Manchester Fungal Infection Group (MFIG), Faculty of Biology, Medicine and Health, University of Manchester, Manchester M13 9NT, UK; jorge.amichelias@manchester.ac.uk

**Keywords:** *Aspergillus fumigatus*, zinc, transcription, regulation

## Abstract

One of the most important features that enables *Aspergillus fumigatus* to grow within a susceptible individual and to cause disease is its ability to obtain Zn^2+^ ions from the extremely zinc-limited environment provided by host tissues. Zinc uptake from this source in *A. fumigatus* relies on ZIP transporters encoded by the *zrfA*, *zrfB* and *zrfC* genes. The expression of these genes is tightly regulated by the ZafA transcription factor that regulates zinc homeostasis and is essential for *A. fumigatus* virulence. We combined the use of microarrays, Electrophoretic Mobility Shift Assays (EMSA) analyses, DNase I footprinting assays and in silico tools to better understand the regulation of the homeostatic and adaptive response of *A. fumigatus* to zinc starvation. We found that under zinc-limiting conditions, ZafA functions mainly as a transcriptional activator through binding to a zinc response sequence located in the regulatory regions of its target genes, although it could also function as a repressor of a limited number of genes. In addition to genes involved in the homeostatic response to zinc deficiency, ZafA also influenced, either directly or indirectly, the expression of many other genes. It is remarkable that the expression of many genes involved in iron uptake and ergosterol biosynthesis is strongly reduced under zinc starvation, even though only the expression of some of these genes appeared to be influenced directly or indirectly by ZafA. In addition, it appears to exist in *A. fumigatus* a zinc/iron cross-homeostatic network to allow the adaptation of the fungus to grow in media containing unbalanced Zn:Fe ratios. The adaptive response to oxidative stress typically linked to zinc starvation was also mediated by ZafA, as was the strong induction of genes involved in gliotoxin biosynthesis and self-protection against endogenous gliotoxin. This study has expanded our knowledge about the regulatory and metabolic changes displayed by *A. fumigatus* in response to zinc starvation and has helped us to pinpoint new ZafA target genes that could be important for fungal pathogens to survive and grow within host tissues and, hence, for virulence.

## 1. Introduction

Zinc is an essential nutrient element that plays a critical role in many different biological processes as a structural and/or as a catalytic component of many different enzymes [1,2]. Indeed, zinc is required, in both pathogens and their hosts, for the normal functioning of hundreds of enzymes and regulatory proteins in all cells. For this reason, zinc uptake and distribution are subjected to a strict homeostatic control. This is critical to ensure an appropriate steady supply of zinc and to prevent the noxious effects that both excess and deficiency of zinc may have on cell growth and differentiation.

Free-living, mutualist and commensal microorganisms obtain zinc from non-living matter such as water, soil or organic compounds present in their surrounding environment. In contrast, microbial pathogens have to obtain zinc from the tissues of the host they parasitize. However, zinc in living tissues is subjected to a strict homeostatic control that restricts the access of pathogens to it. Indeed, intracellular eukaryotic zinc proteins bind zinc tightly with p*K*_d_ stability constants (i.e., −log*K*_d_) of 10–12 [3]. As a consequence, although the concentration of total zinc within most eukaryotic cells is in the micromolar range (100–500 µM) [4,5], the amount of intracellular free Zn^2+^ ions is commensurately about six to seven orders of magnitude lower than the overall cellular zinc concentration. It has been estimated that the concentration of free Zn^2+^ ions in different types of mammalian cells and tissues range from tens to hundreds of picomoles per liter [6]. For instance, the concentration of free Zn^2+^ ions reported in human red blood cells is 24 pM [7], which indicates that a single red blood cell (90 fL volume) should have 1–2 atoms of free Zn^2+^ ions [8].

*Aspergillus fumigatus* is a saprophyte filamentous fungus that usually grows on organic decaying matter. However, it is also able to grow as a parasite in the lungs of immunosuppressed individuals and cause invasive pulmonary aspergillosis (IPA), which is one of the fungal infectious diseases with the highest mortality rate among immunosuppressed patients [9]. One of the most important features that enables *A. fumigatus* to grow within a susceptible host and causes disease is its ability to obtain Zn^2+^ ions from the hostile environment provided by host tissues [10]. *A. fumigatus* zinc uptake from host tissues relies on transporters of the ZIP family encoded by the *zrfA*, *zrfB* and *zrfC* genes [11,12]. The ZrfC transporter seems to be well adapted to function under the alkaline zinc-limiting conditions of mammals’ bodies and plays a major role in zinc uptake from host tissues. Interestingly, the ZrfA and ZrfB zinc transporters, despite their expression are higher in acidic than in alkaline zinc-limiting media, also play a relevant role in virulence. Thus, the virulence of a ∆*zrfA*∆*zrfB*∆*zrfC* mutant is fully abrogated in a murine model of invasive pulmonary aspergillosis [13], as is that of a ∆*zafA* mutant [14], which lacks the gene encoding the zinc-responsiveness transcriptional factor ZafA that regulates zinc homeostasis in *A. fumigatus*. Hence, the ZafA-mediated induction of the zinc uptake system under zinc-limiting conditions is a key homeostatic function required for optimal fungal growth within host tissues.

**Figure 1:**
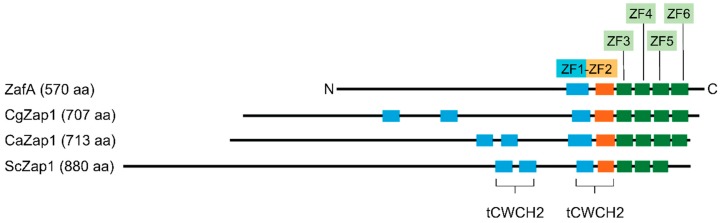
Schematic representation of the transcriptional regulators of zinc homeostasis from *Aspergillus fumigatus* (ZafA), *Cryptococcus gattii* (CgZap1), *Candida albicans* (CaZap1) and *Saccharomyces cerevisiae* (ScZap1). Green boxes indicate canonical zinc finger (ZF) domains of the C2H2-type. Blue and orange boxes indicate ZFs of the CWCH2-type. These ZFs are typically arranged in pairs forming tandem CWCH2 motifs (tCWCH2). The ZF of the CWCH2-type that is presumably able to bind DNA is indicated as an orange box. Protein sequences were aligned taking as reference the ZF of the C2H2-type located towards the N-terminus in ZafA (i.e., ZF3).

The most investigated ZafA orthologue is the Zap1 transcription factor of *Saccharomyces cerevisiae* [15]. Several genome-wide transcriptional profiling studies in *S. cerevisiae* have shown that Zap1 influences the expression of many genes required to implement properly the adaptive and homeostatic responses to zinc deficiency [16,17,18]. Similar studies on Zap1-regulated genes have been also performed in the pathogenic yeasts *Candida albicans* and *Cryptococcus gattii* [19,20]. These Zap1-like yeast transcription factors have 3–4 canonical zinc fingers domains (ZFDs) of the C2H2-type and four ZFDs of the CWCH2-type. The latter are typically clustered in pairs forming two tandem motifs (tCWCH2) [21] (Figure 1). Interestingly, in transcription factors carrying tCWCH2 motifs, only the CWCH2 zinc finger of the tCWCH2 motif closer to a canonical C2H2 zinc finger interacts with the DNA backbone, whereas its partner CWCH2 zinc finger is dedicated to protein-protein interactions and does not interact with the DNA [22,23,24]. Actually, it has been proposed that the tCWCH2 motifs are involved in inter-ZFD interactions and influence both the recognition and binding capacities of their neighboring canonical C2H2 domains to DNA [21]. Since the activity of the transcription factors having tCWCH2 motifs appear to be largely influenced by this type of ZFDs, it is very likely that the transcriptional profile under zinc starvation in yeast carrying Zap1-like factors with two tCWCH2 motifs differs to a certain extent from that observed in *A. fumigatus* and other filamentous fungi carrying ZafA-like factors with a unique tCWCH2 motif [14] (Figure 1). To discover new ZafA target genes essential for fungal virulence other than those encoding ZIP transporters, we performed a genome-wide transcription profiling in *A. fumigatus* grown in vitro under alkaline zinc-limiting conditions to mimic those found by the fungus in the lungs of a susceptible host. In addition, we analyzed the promoter regions of the genes that exhibited the highest differential expression levels between zinc-replete and zinc-limiting conditions. To this purpose, we used a combination of in vitro and in silico procedures to identify the consensus DNA zinc response motif to which ZafA binds under zinc-limiting conditions.

## 2. Materials and Methods

### 2.1. Strains and Culture Media

The *A. fumigatus* strains used in this study are listed in Table 1. These fungal strains were routinely grown in either the PDA complex medium (20 g/L potato dextrose agar, 20 g/L sucrose, 2.5 g/L MgSO_4_-7H_2_O) or in the liquid synthetic dextrose nitrate zinc-limiting medium (SDN–Zn, pH 7.2) (1.7 g/L YNB without amino acids, without ammonium sulphate and without zinc [CYN2401, Formedium], 20 g/L Dextrose, 3 g/L NaNO_3_, 12 µM FeSO_4_-7 H_2_O, 6 µM CuSO_4_-5 H_2_O, 10 µM Na_2_MoO_4_-2 H_2_O), which was supplemented with zinc as specified using a sterile stock solution of 1 mM ZnSO_4_-7H_2_O in ultrapure water.

### 2.2. Culture of Aspergillus fumigatus for RNA Isolation

To obtain RNA under zinc-limiting culture conditions to be used for both microarray analyses and Real Time quantitative PCR (RT-qPCR) experiments, the wild-type and ∆z*afA* mutant strain were inoculated to a density of 1.5 × 10^6^ spores/mL in 200 mL of the SDN–Zn zinc limiting medium and incubated at 37 °C with shaking at 200 rpm for 20 h. Similarly, to obtain RNA under zinc-replete culture conditions, the wild-type strain was inoculated and incubated as described previously in the SDN–Zn zinc limiting medium supplemented with 100 µM zinc. In all cases, spores grown in PDA were used as inoculum.

The mycelia were harvested by filtration on filter paper, washed twice with sterile water and snap-frozen in liquid nitrogen. After grinding the mycelia in the presence of liquid nitrogen, the RNA was extracted using the RNeasy Plant Mini Kit (74904, QIAGEN, Hilden, Germany) according to manufacturer’s instructions. RNA was eluted in 50 µL of RNase-free water. The concentration and quality of RNA was determined by UV spectrometry (Nanodrop ND1000 spectrophotometer, Thermo Fisher Scientific, Waltham, MA, USA) and checked on 0.8% agarose gels stained with ethidium bromide. RNA samples were stored at −80 °C until use.

### 2.3. Microarray Experiments

The *A. fumigatus* Af293 DNA microarray slides (version 4) with 2× replicate 70-mer oligonucleotide printings used in this study were obtained (April 2010) through the Pathogen Functional Genomics Resource Center of the National Institute of the Allergy and Infectious Diseases/National Institute of Health (NIAID/NIH), managed and funded by the Division of Microbiology and Infectious Diseases (DMID) and operated by the J. Craig Venter Institute.

Total RNA was treated with RNase DNase-free and 2 µg of total RNA DNA-free was used for cDNA synthesis, which was performed using SuperScript III RT and a dNTP/5-(3-aminoallyl)-dUTP labelling mix. Upon elimination of unincorporated aa-dUTP, either the Cy5 or Cy3 dye was coupled to the aminoallyl-labelled cDNA by incubation for 1 h at room temperature in 0.1 M sodium carbonate (pH 9.3). Free dyes were removed using the QIAGEN PCR purification kit and were calculated for the total picomoles of dyes incorporation and the pmol cDNA/pmol Cy dye ratios upon measuring spectrophotometrically the cDNA concentration, OD_650 nm_ for Cy5 and OD_550 nm_ for Cy3. Microarray experiments were performed in duplicate with dye labels reversed. Each slide was hybridized with 1:1 mix of two differentially labeled probes Cy3 and Cy5 (1100 pmoles of each one) in a HS 4800 Pro™ Hybridization Station (Tecan Group Ltd., Männedorf, Switzerland) and scanned using the Axon GenePix 4000B microarray scanner. The TIFF images generated were analyzed using Spotfinder 321a to obtain relative transcript levels. Data were normalized with MIDAS using LOWESS (Locally Weighted Scatterplot Smoothing) and the dye-swap procedure (all softwares were developed by the J. Craig Venter Institute to be used for DNA microarray slides provided by the NIAID/NIH). The resulting data were averaged from duplicated genes on each array and from duplicate flip-dye arrays for each experiment, taking a total of four intensity data points for each gene. A volcano-plot algorithm was used to identify genes that exhibited statistical significance (*p* < 0.05) with a change in expression levels ≥ 1.5-fold. Differentially expressed genes at the 95% confidence level were determined using T-test implemented in the MEV program of the TM4 microarray software suite (http://www.tm4.org). The microarray data have been deposited in the GEO database under accession number GSE109389.

### 2.4. RT-qPCR

The concentration of total RNA in all samples were brought to a final concentration of 150 ng/µL. To remove genomic DNA, 1.5 µg of total RNA were treated with RQ1 DNase I (M610, Promega, Madison, WI, USA) and checked by conventional PCR for the complete absence of gDNA. Next, 1 µg of DNase-treated RNA was reversed transcribed using the SuperScript II Reverse Transcriptase (18064-014, Invitrogen, ThermoFisher Scientific) and oligo (dT)_15_ (C1101, Promega) or random hexamers (SO142, ThermoFisher Scientific) as primers. Prior to the qPCR reactions, the cDNA samples were diluted 1:3 in water (except for reactions against the 18S rRNA that were diluted 1:1200 in water). Quantitative real time PCR (qPCR) reactions were performed in a BioRad CFX96 equipment. A typical qPCR reaction mixture (10 µL) contained 13.5 ng cDNA (32 pg when the qPCR was for 18S rRNA), a specific pair of primers (150 nM final concentration) and the SYBR Premix ExTaq (Tli RNaseH Plus) (RR420A, Takara). The amount of cDNA used per reaction was calculated on the basis that all RNA had been reversed transcribed into cDNA, including the rRNAs. Primers used for qPCR are listed in Table S1. PCRs were carried out for 40 cycles, denaturation at 95 °C for 10 s, annealing at 59 °C for 20 s and extension at 72 °C for 20 s. The relative expression level with respect to 18S rRNA (REL/18S) was calculated by the 2^−∆Ct^ method. The relative expression ratio (rER) was calculated by the 2^−∆∆Ct^ method using the expression level of the 18S rRNA as internal reference.

### 2.5. Expression and Purification of the Recombinant *ZafA* Protein

An *Eco*RI-flanked DNA fragment (482 bp) carrying the coding sequence for the C-terminal 140 amino acids of ZafA (residues 434–570) was obtained by high-fidelity PCR using as template the plasmid pZAF14, which carried the complete cDNA for the ZafA coding sequence [14], and the pair of oligonucleotides JA181/JA182 (Table S2). This fragment was digested with *Eco*RI and ligated to the *Eco*RI site of the pGEX-5X-3 plasmid (GE Healthcare, Little Chalfont, UK) to generate the pGEX-5X-ZafA plasmid. The ZafA coding sequence inserted into the pGEX-5X-ZafA plasmid was confirmed by sequencing. The pGEX-5X-ZafA plasmid was used to transform *Escherichia coli* BL21-DE3. The resulting strain was able to synthesize high amounts of the four C-terminal zinc fingers of ZafA (ZF3-6) fused to the C-terminus of GST upon induction with IPTG (0.25 mM). Although the GST-ZafA^ZF3-6^ protein aggregated into inclusion bodies, it was easily solubilized using a slightly acidic lysis buffer (20 mM Tris-HCl, 150 mM NaCl, 100 mM ZnCl_2_, 0.1% Triton X-100, 5 mM DTT [pH 6.7]), instead of the most standard lysis buffer at pH 7.5. The GST-ZafA^ZF3-6^ protein was purified using Glutathione Sepharose 4B (GE Healthcare, Cat. 17075601) and eluted with elution buffer (50 mM Tris-ClH [pH 8.0], 150 mM NaCl, 0.1% Triton X-100, 10 mM reduced glutathione). The eluted GST-ZafA^ZF3-6^ protein was concentrated with an Amicon Ultra-15 3K device (Millipore, Burlington, MA, USA) and dialyzed in a D-Tube Dialyzer Maxi unit (MWCO 3.5 kDa) (Merck-Millipore, Novagen, Temecula, CA, USA) against the factor Xa buffer (20 mM Tris-Cl, pH 6.5, 50 mM NaCl, 1 mM CaCl_2_). Upon optimizing the cleavage conditions of the GST-ZafA^ZF3^^-6^ protein with factor Xa, the purified GST-ZafA^ZF3-6^ protein was treated with 25 ng factor Xa (NEB, P8010, Ipswich, MA, USA) per 260 ng of protein for 6 h at room temperature to achieve 100% cleavage of GST-ZafA^ZF3-6^ protein, as judged in SDS-PAGE gels stained with coomassie blue. The factor Xa was removed from the cleavage reaction with Xarrest™ Agarose (Merck-Millipore, cat. 69038). Following the capture of Factor Xa, the agarose was removed by spin-filtration. The cleavage reaction without factor Xa, which contained equimolar amounts of GST and ZafA^ZF3-6^, was dialyzed against binding buffer (25 mM Tris-HCl, pH 8.0, 50 mM KCl, 10 μM ZnCl_2_, 1 mM MgCl_2_, 1 mM DTT). Proteins were stored at −20 °C until use.

The GST protein that was used as control was expressed from plasmid pGEX-5X-3, purified, digested with factor Xa and dialyzed as described for the GST-ZafA^ZF3-6^ protein.

Protein concentrations were estimated by measuring A280 nm in a NanoDrop spectrophotometer (ThermoFisher) and taking into consideration that the extinction molar coefficients for the GST and ZafA^ZF3-6^ moieties of the GST-ZafA^ZF3-6^ protein were 1.632 and 0.1811 mg^−1^ × mL × cm^−1^, respectively.

### 2.6. Construction of Plasmids to Obtain Probes for Electrophoretic Mobility Shift Assays and DNase I Footprinting Experiments

Two overlapping DNA fragments of the *zafA* promoter (P*zafA*) of 397 bp and 233 bp were obtained by PCR using as template gDNA from the wild-type AF14 strain and the pair of oligonucleotides JA204/JA394 and JA375/JA204, respectively (Table S2). The PCR products were ligated to the pGEM-T vector (Promega, cat. A1360) to generate the pZAF151 and pZAF91 plasmids.

The complete sequence of the bidirectional promoter P*aspf2-zrfC* that drives the expression of the *aspf2* and *zrfC* genes (abbreviated as P*zrfC*, 884 bp) was amplified by PCR using as template gDNA from the wild-type AF14 strain and the pair of oligonucleotides JA194/JA196. The PCR product was ligated to the pGEM-T easy vector (A1360, Promega) to generate the pASFP2-ZRF31 plasmid. Similarly, a 215-bp DNA fragment of P*zrfC* right next to the *zrfC* coding sequence was amplified using the pair of oligonucleotides JA376/JA377 and the PCR product was ligated to the pGEM-T vector to generate the pASFP2-ZRF311 plasmid. Finally, 539 bp of P*zrfC* right next to the *zrfC* coding sequence were excised from the pASFP2-ZRF31 plasmid following an *Eco*RV/*Msc*I digestion-religation to generate the pASFP2-ZRF312 plasmid, which only carried 349 bp of P*zrfC* right next to the *aspf2* coding sequence.

A 222-bp DNA fragment of the *zrfA* promoter (P*zrfA*) was amplified by PCR using as template gDNA from the wild-type AF14 strain and the pair of oligonucleotides JA378/JA379. The PCR product was ligated to the pGEM-T vector to generate the pZRF10 plasmid.

A 256-bp DNA fragment of the *zrfB* promoter (P*zrfB*) was amplified by PCR using as template gDNA from the wild-type AF14 strain and the pair of oligonucleotides JA380/JA381. The PCR product was ligated to the pGEM-T vector to generate the pZRF261 plasmid.

A 191 bp-DNA fragment of the *alcA* promoter of *Aspergillus nidulans* (P*alcA*) was amplified by high-fidelity PCR using the pair of oligonucleotides JA261/JA388 and as template gDNA from the *A. nidulans* strain G1059 [25]. The PCR product was ligated to the pGEM-T vector to generate plasmid pALC3.

All subcloned PCR products were sequenced to verify the absence of any mutation that could have been introduced during PCR.

### 2.7. Preparation of Probes for Electrophoretic Mobility Shift Assays

DNA probes for different promoters (P*zafA*, P*zrfC*, P*zrfA*, P*zrfB* and P*alcA*) were amplified by high-fidelity PCR using Pfu polymerase and purified using the QIAquick PCR Purification Kit (QIAGEN). The oligonucleotides used as primers (Table S2) and plasmids used as templates for DNA amplification by PCR were the following: For P*zafA* EMSA assays we used a 233-bp DNA fragment amplified by PCR using a dilution of the pZAF91 plasmid as template and the pair of oligonucleotides JA375/JA204 as primers; For P*zrfC* EMSA assays we used a 221-bp DNA fragment amplified by PCR using a dilution of the pASFP2-ZRF311 plasmid as template and the pair of oligonucleotides JA376/JA377; For P*zrfA* EMSA assays we used a 222-bp DNA fragment amplified by PCR using a dilution of the pZRF10 plasmid as template and the pair of oligonucleotides JA378/JA379 as primers; For P*zrfB* EMSA assays we used a 256-bp DNA fragment amplified by PCR using a dilution of the pZRF261 plasmid as template and the pair of oligonucleotides JA380/JA381 as primers; For P*alcA* EMSA assays we used a 213-bp DNA fragment amplified by PCR using a dilution of the pALC3 plasmid as template and the pair of oligonucleotides JA261/JA388 as primers.

### 2.8. Preparation of Probes for DNase I Footprinting Assays

6-FAM-5′-labelled DNA probes for different promoters (P*zafA*, P*zrfC*, P*zrfA*, P*zrfB* and P*alcA*) were amplified by high-fidelity PCR using Pfu polymerase and purified using the QIAquick PCR Purification Kit (QIAGEN). The oligonucleotides used as primers (Table S2) and plasmids used as templates in DNA amplification by PCR are described next.

Four different probes were used for the P*zafA* DNase I footprinting assays: (i) Two 432 bp DNA fragments amplified by PCR using the pZAF91 plasmid as template and the pair of oligonucleotides FAM-Rv/Fw and Rv/FAM-Fw as primers to get them labelled in 5′-ends of the sense and antisense strands respectively (these probes carried 229 bp of P*zafA* plus two flanking sequences corresponding to the MCS of the vector); (ii) A536 bp DNA fragment amplified by PCR using the pZAF151 plasmid as template and the pair of oligonucleotides FAM-Rv/JA204 as primers to get it labelled in the 5′-end of the sense strand (this probe carried 396 bp of P*zafA* plus one flanking sequence corresponding to the MCS of the vector); (iii) A 435 bp DNA fragment amplified by PCR using the pZAF151 plasmid as template and the pair of oligonucleotides Rv/FAM-JA439 as primers to get it labelled in the 5′-end of the antisense strand (this probe carried 295 bp of P*zafA*).

Four different probes were used for the P*zrfC* DNase I footprinting assays: (i) A 322 bp DNA fragment amplified by PCR using the pASPF2-ZRF311 plasmid as template and the pair of oligonucleotides FAM-Fw/JA377 as primers to get it labelled in the 5′-end of the sense strand for *zrfC* (this probe carried 215 bp of P*zrfC* plus one flanking sequence corresponding to the MCS of the vector); (ii) A 347 bp DNA fragment amplified by PCR using the pASFP2-ZRF311 plasmid as a template and the pair of oligonucleotides JA376/FAM-Rv as primers to get it labelled in the 5′-end of the antisense strand for *zrfC* (this probe carried 215 bp of P*zrfC* plus one flanking sequence corresponding to the MCS of the vector); (iii) A 393 bp DNA fragment amplified by PCR using the pASPF2-ZRF312 plasmid as template and the pair of oligonucleotides FAM-Fw/JA7 as primers to get it labelled in the 5′-end of the sense strand for *zrfC* (this probe carried 349 bp of P*zrfC* plus one flanking sequence corresponding to the MCS of the vector); (iv) A 504 bp DNA fragment amplified by PCR using the pASPF2-ZRF312 plasmid as template and the pair of oligonucleotides JA194/FAM-Rv as primers to get it labelled in the 5′-end of the antisense strand for *zrfC* (this probe carried 349 bp of P*zrfC* plus one flanking sequence corresponding to the MCS of the vector).

Two different probes were used for the P*zrfB* DNase I footprinting assays: (i) A 456 bp DNA fragment amplified by PCR using the pZRF261 plasmid as template and the pair of oligonucleotides FAM-Fw/Rv as primers to get it labelled in the 5′-end of the sense strand; (ii) A 456 bp DNA fragment amplified by PCR using the pZRF261 plasmid as template and the pair of oligonucleotides Fw/FAM-Rv as primers to get it labelled in the 5′-end of the antisense strand. Both probes carried 256 bp of P*zrfB* plus two flanking sequences corresponding to the MCS of the vector.

One probe was used for the P*zrfA* DNase I footprinting assays. A 350 bp DNA fragment amplified by PCR using the pZRF10 as template and the pair of oligonucleotides JA378/FAM-Rv as primers to get it labelled in the 5′-end of the antisense strand (this probe carried 217 bp of P*zrfA* plus one flanking sequence corresponding to the MCS of the vector).

One probe was used for the P*alcA* DNase I footprinting assays. A 413 bp DNA fragment of the pALC3 plasmid carrying 213 bp of P*alcA* plus two flanking sequences corresponding to the MCS of the vector were amplified by high-fidelity PCR and the pair of oligonucleotides FAM-Fw/Rv or Fw/FAM-Rv.

### 2.9. Electrophoretic Mobility Shift Assays and DNase I Footprinting Assays Reactions and Analyses

Reactions for EMSA experiments (20 µL) were assembled in 0.5-mL microtubes and carried out in EMSA buffer (25 mM Tris-HCl [pH 8], 50 mM KCl, 1 mM MgCl_2_, 12.5% glycerol, 1 mM DTT), containing 1 µL BSA (0.2 mg/mL), 0.25–0.75 pmoles of the target DNA and the amount of the equimolar mixture of GST and ZafA^ZF3-6^ required to add 0.125–24 pmoles ZafA^ZF3-6^. Reactions were incubated for 1 h at 30 °C before adding 4 µL of loading buffer (20% sucrose, 0.002% bromophenol blue). Reactions were loaded in a polyacrylamide 5% EMSA gel that had been pre-run in TBE 1× for 10 min at 4 °C in a cold room. Gels were run at 100 V for approximately 75 min, stained with a solution of ethidium bromide (5 µg/mL) for 20 min at room temperature, washed for 10 min with distilled water and photographed on a UV transilluminator in a gel documentation system.

Reactions for footprinting assays (60 µL) were assembled in 0.5-mL microtubes and carried out in an EMSA buffer containing 3 µL BSA (0.2 mg/mL), 0.5 pmoles of the desired 6-carboxyfluorescein (6-FAM)-labeled DNA fragment and 12 pmoles of ZafA^ZF3-6^. Control reactions were assembled identically but omitting ZafA^ZF3-6^. All DNA fragments used for footprinting assays were obtained by high-fidelity PCR using as primers both a 6-FAM-5′-labelled oligonucleotide and a non-labeled oligonucleotide, as described above. After incubating the footprinting reactions for 45 min at 30 °C, DNA was partially digested with 0.01 U of DNase I by adding 4 µL of a DNase I stock solution (0.0025 U/µL) per reaction and incubated at 25 °C for 1 min. The reaction was stopped immediately by adding 340 µL of stop buffer (9 mM Tris-HCl [pH 8], 40 mM EDTA). DNA was extracted with one volume of phenol:chloroform:isoamyl alcohol (25:24:1) followed by an extraction with one volume of chloroform:isoamyl alcohol (24:1). DNA in the aqueous phase was precipitated with 0.1 volumes of a non-buffered 3.0 M AcNa solution plus 2.5 volumes of ethanol. Precipitation of DNA was left to stand to proceed overnight at −20 °C. After centrifugation, the DNA pellet was washed twice with 70% ethanol, allowed to air dry at room temperature, suspended in 10 μL of Hi-Di formamide, denatured at 95 °C for 3 min and cooled on ice. All DNase I footprinting reactions were analyzed electrophoretically on an automated capillary DNA sequencer using the GeneScan^TM^ 500 LIZ (Thermofisher) dye Size Standard as a calibrator. Each footprinting reaction was performed in parallel to four dideoxynucleotide-based sequencing reactions of the corresponding DNA probe, using as a primer the same 6-FAM 5′-labelled oligonucleotides utilized to amplify each DNA fragment by PCR.

### 2.10. Construction of Plasmids Used for Aspergillus fumigatus Transformation

The strains AFZR0 and AFZR1 of *A. fumigatus* used in this study were able to express the coding sequences of both the firefly luciferase (*luc*; as a reporter of *aspf2* expression) and the green fluorescent protein (*gfp*; as a reporter of *zrfC* expression) under control of either a wild-type version of the bidirectional *aspf2-zrfC* promoter (P*zrfC*^wt^) or a mutant version of this promoter whose ZR motifs had been inactivated by site directed mutagenesis (P*zrfC*^ZR123^).

The CEA17 uridine-uracil-auxotrophic *pyrG1* strain was transformed with two different EcoRI-EcoRI DNA fragments of 7286 bp, excised respectively from plasmids pPYRGQ191 and pPYRGQ193 to generate respectively the AFZR0 and AFZR1 strains (Figure S1).

The plasmids pPYRGQ191 and pPYRGQ193 were generated by ligating XbaI-FspI DNA fragments (4105 bp), which carried respectively the [*luc* ← P*zrfC*^wt^ → *gfp*] and [*luc* ← P*zrfC*^ZR123^ → *gfp*] constructs, to the pPYRGQ31 plasmid digested with XbaI/SmaI. The pPYRGQ31 plasmid was an improved version of the pPYRGQ3 plasmid that had been designed previously in our laboratory to revert specifically the *pyrG1* mutation (C756T) in the *A. fumigatus* CEA17 or in any PyrG^–^ CEA17 derivative strain and select PyrG^+^ prototrophic strains bearing the DNA fragment of interest inserted between its AFUA_2G08360 (*pyrG*) and AFUA_2G08350 loci [11].

The plasmids used to transform *A. fumigatus* were linearized by digestion with *Eco*RI, extracted with phenol:chloroform:IAA, precipitated with acetate/isopropanol, washed in 70% ethanol, dissolved in 50% (*v*/*v*) of KC solution (0.6 M KCl, 50 mM CaCl; pH 6.0–6.5) and used for transformation as described below.

### 2.11. Generation of Protoplasts and Transformation of Aspergillus fumigatus

Protoplasts of *A. fumigatus* were prepared using a new protocol developed in our laboratory to obtain high-quality protoplasts that were very susceptible to transformation. In brief, to obtain protoplasts of the CEA17 PyrG^–^ strain we inoculated 5 × 10^8^ conidia of this strain in the SDN medium supplemented with 0.05% (*w*/*v*) uracil, 0.12% (*w*/*v*) uridine and 20% (*v*/*v*) of a sterile conditioned medium that was produced by ourselves and contained a highly active α-glucanase. The culture was incubated at 37 °C for 14 h. Germlings were collected by filtration through a cell strainer unit (40 µm), suspended in 10 mL of protoplasting buffer (0.2 g VinoTaste, 0.75 M KCl, 25 mM citrate/phosphate; pH 5.8) (it had been previously sterilized by filtration through a 0.22 µm filter unit) and incubated at 35 °C with shaking at 120 rpm for 2 h. The protoplasting suspension was filtered through a miracloth (Merck-Millipore, Calbiochem Cat. 475855; 22–25 µm) and centrifuged at 1200× *g* for 10 min a 4 °C. The protoplast pellet was washed in 15 mL of a cold KC solution (0.6 M KCl, 50 mM CaCl; pH 6.0–6.5) by mixing gently and centrifuged at 1200× *g* for 10 min at 4 °C. The protoplast pellet was suspended into 0.4 mL of KC solution and used for transformation.

Aliquots of 0.2 mL of protoplast suspension were dispensed in 15-mL conical tubes to which were added sequentially the transforming DNA (~10 µg) and 0.5 mL of PEG buffer (30% PEG-6000 [*w*/*v*], 0.6 M KCl, 50 mM CaCl_2_, 10 mM Tris-HCl; pH 7.5). The protoplast-DNA-PEG mixture was incubated on ice for 30 min and then at 25 °C for 30 min in a water bath. Next, 9.3 mL of KC solution was added to each protoplast-DNA-PEG solution, mixed by inversion, centrifuged at 1200× *g* for 10 min at 4 °C, suspended in 0.5 mL of KC solution, mixed with 6 mL of soft agar (AMM with 0.6 M KCl plus 0.4% [*w*/*v*] agarose) and poured onto two selective AMM plates. Plates were incubated at 37 °C until PyrG^+^ fungal transformants had grown. Several independent transformants were isolated and re-isolated on AMM agar plates. The transformants that had undergone homologous recombination at the expected locus were confirmed by PCR. A sample of conidia scrapped with a cotton swab from a colony of each isolated was inoculated into 1 mL of AMM and incubated overnight at 37 °C to obtain minipreps of genomic DNA suitable for PCR analysis.

### 2.12. Detection of SOD Activity on PAGE Gels

After 20 h of incubation of a wild-type strain in liquid SDN–Zn medium, mycelium was harvested through filtration, washed with cold sterile water and snap-frozen on liquid N_2_. Mycelium was ground in a mortar with a pestle in presence of liquid N_2_ until it became a fine dust, suspended into 0.4 mL of native protein extraction buffer (36 mM KH_2_PO_4_/K_2_HPO_4_ [pH 7.8]; 1 mM EDTA; 0.1% Triton X-100; 5% [*v*/*v*] glycerol; 0.5% [*v*/*v*] Protease inhibitor cocktail-EDTA [Thermo Scientific, cat. 87785]) and clarified by centrifugation at 14,000 × *g* for 10 min at 4 °C. Proteins were separated by native PAGE (T = 8%) at 120 V at 4 °C and gels were washed twice with distilled water (10 min each). Gels were incubated for 20 min in 50 mL of an Nitro Blue Tetrazolium Chloride (NBT) solution (2.45 mM [N6876, Sigma-Aldrich, St. Louis, MO, USA] in 36 mM KH_2_PO_4_/K_2_HPO_4_ [pH 7.8]), transferred to the developing solution (28 µM riboflavin, 28 mM TEMED in 36 mM KH_2_PO_4_/K_2_HPO_4_ [pH 7.8]) and illuminated with white light until transparent bands were readily observed in a dark-blue background due to formazan generated after reduction of NBT with O_2_^−^ formed by reduction of riboflavin in the presence of O_2_.

## 3. Results

### 3.1. Under Zinc-Limiting Conditions *ZafA* Influences the Expression of Genes Related to Many Different Metabolic and Biosynthetic Processes in Addition to Those Required to Maintain Zinc Homeostasis

When *A. fumigatus* grows under zinc-limiting conditions, a significant change in the transcription level of the most direct ZafA-target genes is expected. In addition, other genes not targeted by ZafA may also change their expression levels as a side effect of the adaptation to the zinc-limiting conditions triggered by ZafA. To identify all the putative ZafA-target genes we designed an experimental approach based in the following theoretical assumptions: (i) Some genes that changed their expression level in a wild-type strain under zinc-limiting conditions would be regulated either directly or indirectly by ZafA, whereas other genes would change their expression level in response to zinc starvation in a ZafA-independent manner. We referred to the former genes as putative directly and indirectly ZafA-regulated (DZR and IZR) genes, whereas the later ones were referred to as non-ZafA-regulated (NZR) genes; (ii) The NZR genes could be either up-regulated or down-regulated both in the wild-type strain and Δ*zafA* mutant grown under zinc-limiting with respect to zinc-replete conditions; (iii) The expression level of the DZR genes in a Δ*zafA* strain should be similar under both zinc-limiting and zinc-replete conditions; (iv) The intrinsic lack of the appropriate homeostatic and adaptive responses to zinc starvation in the ∆*zafA* strain would result in a strain-specific adaptive response to relieve the lack of *zafA* in this mutant that might involve changes in the expression level of many genes that in a wild-type strain would not change under zinc starvation, and that we referred to as strain-specific adaptive (SSA) genes.

**Figure 2:**
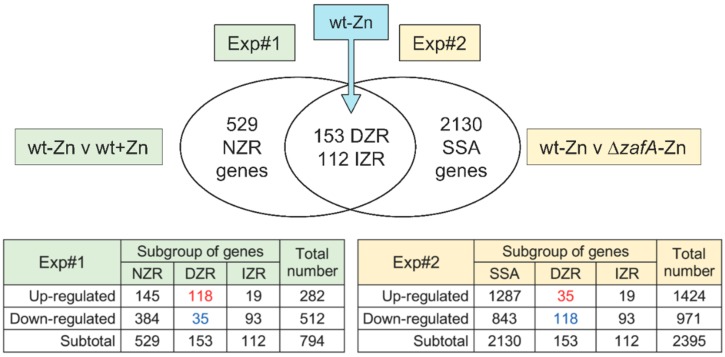
Quantitative summary of different types of genes detected in microarray experiments. SSA: strain-specific adapted (genes); DZR: directly ZafA regulated; IZR: indirectly ZafA regulated; NZR: non-ZafA regulated.

Therefore, by keeping these assumptions in mind we performed two independent microarray experiments (Exp#1 and Exp#2) (Figure 2). Exp#1 was aimed to determine genes expressed differentially in a wild-type strain grown under zinc-limiting conditions versus zinc-replete conditions (Results in Table S3). Exp#2 was aimed to determine the genes expressed differentially in a Δ*zafA* strain with respect to a wild-type strain, both grown under zinc-limiting conditions (Table S4). Upon comparing the differentially regulated genes detected in Exp#1 and Exp#2, 112 putative IZR genes (19 up-regulated and 93 down-regulated in both the wild-type strain and Δ*zafA* mutant) and 153 putative DZR genes were identified (Figure 2). More specifically, 118 DZR genes that were up-regulated in the wild-type strain according to Exp#1 emerged as down-regulated in the ∆*zafA* strain according to Exp#2, whereas 35 DZR genes that were down-regulated in the wild-type strain according to Exp#1 emerged as up-regulated in the ∆*zafA* strain according to Exp#2 (Table S5). It would be expected that the direct targets of ZafA were included among the DZR genes. It would be expected that the genes targeted indirectly by ZafA were included among the IZR genes.

To validate the microarray analysis, we measured by RT-qPCR the relative expression ratio of 56 genes whose expression changed significantly in a wild-type strain grown under zinc-limiting versus zinc-replete conditions. More precisely, we measured the relative expression of 10 IZR, 22 NZR and 24 DZR genes (Table 2). The expression levels of the *zafA*, *zrfA*, *zrfB* and *zrfC* genes, whose expression profiles had been well defined previously [11,12,14], were measured as positive controls. The expression of the actin gene (AFUA_6G04740/*actA*), whose expression did not change significantly under zinc starvation neither in a wild-type nor in a Δ*zafA* mutant [11,14], was measured as a negative control. In addition, the expression levels of six additional genes selected at random, whose expression did not change also under zinc starvation neither in a wild-type nor in a Δ*zafA* mutant, were also measured as negative controls (Table 2). The results obtained by both techniques were coincident and the correlation between microarray data and those measured by RT-qPCR were highly significant (Pearson’s *r* = 0.69, *P* < 0.0001) in spite of the high difference in sensitivity and accuracy between these experimental procedures.

### 3.2. Zinc Starvation Influences the Expression of Genes Related to Many Different Biological Processes

A functional categorization of the 512 down-regulated and 282 up-regulated genes in a wild-type strain grown under zinc-limiting conditions was performed on the *Aspergillus fumigatus* AF293 (AspGD) genome using the gene ontology (GO) tool integrated in FungiFun2, which is an updated and easy-to-use online resource for functional enrichment analysis of fungal genes [26]. The functional categorization of the down-regulated genes revealed that 26.0% of the input genes were significantly categorized (i.e., 133/512). These genes were related to ergosterol biosynthesis (*p* = 1.32 × 10^−9^), cellular response to iron starvation (*p* = 1.49 × 10^−9^), fatty acid biosynthesis (*p* = 3.65 × 10^−5^), α(1,3)-glucan biosynthesis (*p* = 0.00013), calcium transport (*p* = 0.00013) and cellular response to hypoxia (*p* = 0.0005). The functional categorization of the up-regulated genes revealed that 18.8% of the genes were significantly categorized (i.e., 53/282). These genes were related to ascospore formation (*p* = 1.74 × 10^−5^), asexual development (conidia formation) (*p* = 2.08 × 10^−4^), mitochondrial respiratory chain complex I (*p* = 8.5 × 10^−5^), zinc transport (*p* = 8.5 × 10^−5^), cellular hyperosmotic response (*p* = 0.00021), and G1 cell cycle arrest in response to nitrogen starvation (*p* = 0.00078). Hence, by using FungiFun2 the functional categorization of 186 genes could be described. In a more careful manual revision we were able to functionally allocate 291 genes into 28 different biological processes and outline the major homeostatic and adaptive responses displayed by the fungus to deal with zinc starvation (Table S6) (see discussion). 247 genes encoded uncharacterized proteins that either were similar to characterized proteins in other organisms or harbored at least one catalytic domain with a predicted function, regardless of its putative subcellular location. Finally, there were also 256 genes that encoded either uncharacterized protein orthologues in other organisms or hypothetical proteins.

Thus, it became apparent that many genes were down regulated under zinc-limiting conditions, mainly those whose expression was induced under iron starvation or influenced by the intracellular calcium levels, those required for fatty acid and phospholipid biosynthesis, and those encoding enzymes involved in ergosterol biosynthesis and whose expression was also strongly co-regulated by iron and hypoxia. Other down-regulated genes encoded proteins related to ethanol metabolism, disulfide bond formation in the endoplasmatic reticulum (ER), enzymes of the citric acid cycle and glycolytic pathway, and components of the mitochondrial electron transport chain (ETC) (Table S6). On the other hand, it was overwhelming the predominance, under zinc-limiting conditions, of up-regulated genes involved in sexual and asexual development, hyphal growth and autophagy, ribosomal assembly and/or translation, response to hyperosmotic stress and regulating and maintaining zinc homeostasis (Table S6). Zinc starvation also influenced, either positively or negatively, the expression of genes that were typically regulated in response to hypoxia and oxidative stress. In addition, the expression of genes involved in biosynthesis of the cell wall, growth factors (riboflavin, thiamine and pyridoxin), heme group, detoxification of D-2-hydroxyglutarate coupled to D-lactate formation, acetate utilization, cytoskeleton and chromatin remodeling and regulation of the cell cycle were also influenced by zinc starvation. Finally, zinc starvation also noticeably influenced the expression levels of genes involved in NAD, sulfur, spermidine and nitrogen metabolism, and others involved in biosynthesis of some secondary metabolites (Table S6). In this regard, it was remarkable that the expression of genes related to gliotoxin biosynthesis was strongly induced under zinc-limiting conditions.

The analysis of the distribution of the 291 genes, categorized functionally based on the most likely role of ZafA in regulating their expression, as defined by their assignation to either the DZR/IZR or NZR group of genes (Table S6), indicated that ZafA influenced either directly or indirectly the expression of ≥60% of the genes involved in the homeostatic response to zinc starvation, fungal development, response to oxidative stress, nitrate assimilation, acetate utilization and in the functioning of the tricarboxylic acid cycle and mitochondrial electron transport chain.

### 3.3. The *ZafA* Transcription Factor Binds to Promoter Regions of Homeostatic Genes Whose Transcription Is Induced under Zinc-Limiting Conditions

**Figure 3:**
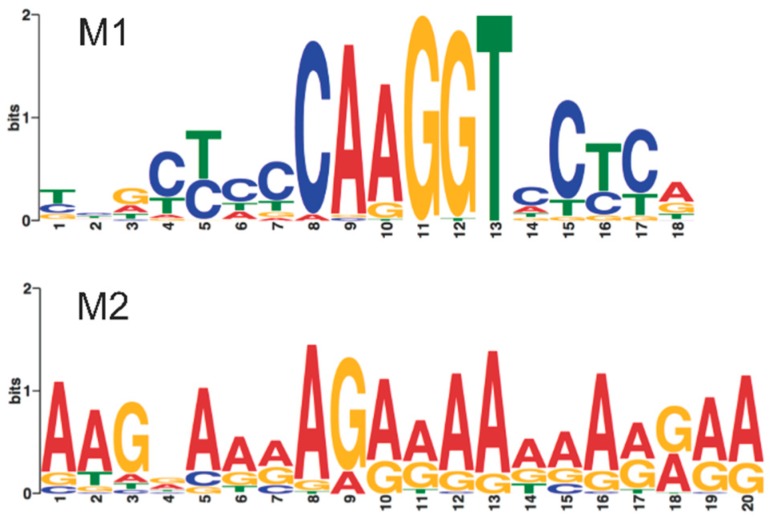
Overrepresented motifs in the promoter regions of the most DZR genes. These motifs were found using the MEME algorithm after analyzing DNA sequences expanding from −20 to −1000 bp from the predicted translation start codons of the ORFs of 67 DZR genes (for numbering purposes, we assigned position −1 to the nucleotide preceding the A of the ATG start codon).

We assumed that the DZR genes should be the ones most directly targeted by ZafA, such that in their promoter regions there would be at least a copy of a zinc responsive (ZR) consensus sequence to which ZafA might bind. Hence, we used the MEME algorithm as a first approach to identify potential regulatory elements and/or repeated patterns shared among the promoters of 67 genes that could be the most direct ZafA target genes [27]. These genes were chosen on the basis of their fold changes in Exp#1, such that the 54 genes more up-regulated and the 13 genes more down-regulated were selected (Table S5). Thus, we found two motifs (M1 and M2) that were found significantly distributed among the analyzed promoters (E-value for M1 = 3.6 × 10^−37^; E-value for M2 = 1.7 × 10^−17^) (Figure 3). The M1 motif showed two short CT-rich sequences flanking the 5′-CAAGGT-3′ core sequence and was present in a number of 1 to 5 copies in the promoter regions of 28 genes (i.e., 42.4% of the analyzed genes). The M2 motif corresponded to a relatively homogeneous long AG rich sequence and was present in a number of 1 to 6 copies in the promoters of 36 genes (i.e., 54.5% of the analyzed genes). However, a control MEME search for repeated patterns in the promoter regions of 71 genes (38 SSA genes plus 33 genes whose expression was not influenced by zinc starvation that were selected at random) (Table S7), also produced a M2-like AG-rich motif in 52.1% of the genes (E-value for the M2-like motif = 3.9 × 10^−59^). Besides, the M2 motif was not detected in the promoter region of the genes *zrfA* and *zrfC*, whose transcription had been shown to be strongly and directly up-regulated by ZafA under zinc-limiting conditions [11,14]. In contrast, the M1 motif was found in a number of 3 to 5 copies in the promoter regions of the *zafA*, *zrfA*, *zrfB*, *zrfC* and *zrfF* genes, that encoded the major components of the zinc homeostatic system in *A. fumigatus*. Moreover, by using the RSAT algorithm [28], we found that one motif nearly identical to M1 was widely distributed among promoters of all DZR genes, whereas no motif detected among IZR genes showed any similarity with M1. Taken together, these findings suggested that the M1 motif could be a genuine DNA regulatory sequence for binding ZafA.

**Figure 4:**
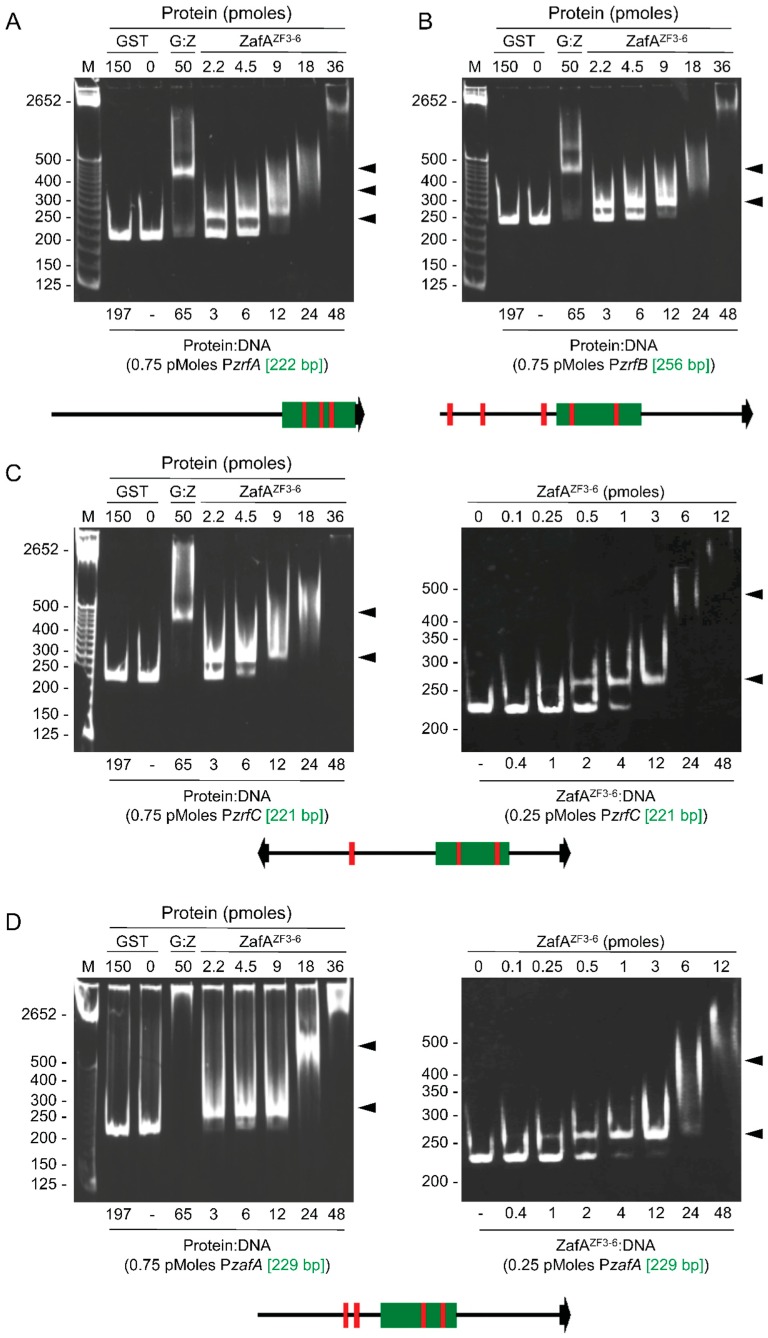
Analyses by Electrophoretic Mobility Shift Assays (EMSA) of the interaction of ZafA^ZF3-6^ with different DNA fragments. (**A**) Interaction of ZafA^ZF3-6^ with a DNA fragment from a promoter region of *zrfA* (green box) that carried three putative ZafA binding motifs (red boxes); (**B**) interaction of ZafA^ZF3-6^ with a DNA fragment from a promoter region of *zrfB* (green box) that carried two putative ZafA binding motifs (red boxes); (**C**) interaction of ZafA^ZF3-6^ with a DNA fragment from a promoter region of *zrfC* (green box) that carried two putative ZafA binding motifs (red boxes); (**D**) interaction of ZafA^ZF3-6^ with a DNA fragment from a promoter region of *zafA* (green box) that carried two putative ZafA binding motifs (red boxes). In the right panel of (C) and (D), reactions were analyzed with lower ZafA^ZF3-6^:DNA ratios to gain resolution. All DNA fragments were also incubated without protein and in the presence of both 150 pmoles of purified GST and 50 pmoles of the fusion protein GST-ZafA^ZF3-6^ (as controls) to attain a GST:DNA ratio of 197 and a GST-ZafA^ZF3-6^:DNA (G:Z) ratio 65. A 25-bp DNA ladder was used as a reference.

To show whether ZafA was able to bind to the promoter regions of the homeostatic genes *zafA*, *zrfA*, *zrfB* and *zrfC*, we analyzed by EMSA the ability of ZafA to bind to short DNA fragments (210–260 bp) of these promoters harboring the previously identified M1 motif. All attempts to express the full-length ZafA protein in *E. coli* were unsuccessful. However, we had success in expressing a recombinant polypeptide corresponding to the C-terminal 140 amino acids of ZafA (residues 434–570 that included the four most C-terminal zinc fingers ZF3-6) fused to the C-terminus of GST. By using this polypeptide we confirmed the binding and observed that the mobility of all DNA-protein complexes in PAGE gels reduced stepwise as the ZafA:DNA ratio increased, which indicated that ZafA^ZF3-6^ was able to bind, with different affinities, to more than one ZafA binding site in all DNA fragment tested (Figure 4). Particularly, three steps were observed for the P*zrfA* DNA fragment (Figure 4A) and two for the P*zrfB*, P*zrfC* and P*zafA* DNA fragments (Figure 4B and left panels of C and D), which matched the number of M1-like motifs found in these promoters. Besides, given that at a ZafA:DNA molar ratio of 6.0 both the P*zrfC* and P*zafA* fragments were almost completely shifted (Figure 4C,D, left panels), we performed EMSAs assays at lower ZafA:DNA ratios (between 0.4 and 4.0, right panels). As shown, in both cases, a ZafA:DNA ratio of four was sufficient to shift most of the DNA. This suggested that in both fragments P*zafA* and P*zrfC* there were two ZR motifs to which two ZafA molecules would bind with similar affinities but stronger than those used to bind to the ZR motifs in the P*zrfA* and P*zrfB* fragments.

### 3.4. The *ZafA* Transcription Factor Binds Specifically to a 15-bp Zinc-Response Consensus Sequence

**Figure 5:**
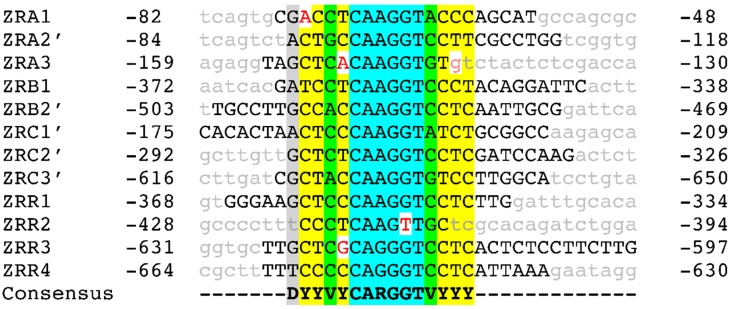
Alignment of DNA sequences protected against DNase I digestion from the DNA fragments used for EMSA analyses. Protected sequences are in capital letters whereas the surrounding sequences are indicated in gray lower case letters (D = A or G or T; V = A or G or C; R = A or G). ZR motifs labelled with a prime symbol indicate that the DNA sequence shown in the picture corresponded to that in the antiparallel strand.

To identify the DNA sequence to which ZafA binds, we performed a fluorescent DNase I footprinting analysis by using fluorescently 6-FAM-labeled primers in parallel to dideoxynucleotide DNA sequencing. This procedure allows the accurate separation of 5′-end-labeled DNA fragments using a capillary-based automated DNA sequencer and to identify the nucleotide sequence of sites protected against DNase digestion [29]. To identify the DNA binding site for ZafA we used several 6-FAM-5′-labelled DNA probes carrying short fragments of the promoter regions from the homeostatic genes *zrfA*, *zrfB*, *zrfC* and *zafA* (see material and methods section) that harbored 1–2 copies of the M1 motif detected previously by MEME analyses. Thus, in the P*zafA* fragments used as probes, four different regions protected by ZafA from its enzymatic digestion with DNase I were found, that were called ZRR1-4 (for Zinc-Response motif located in the promoter region of gene encoding the Regulatory protein ZafA) (Figure 5 and Figure S2). Similarly, in the P*zrfA* and P*zrfC* fragments used as probes, three different ZafA-protected regions in each one were found, that were called ZRA1-3 and ZRC1-3 (for Zinc-Response motif located respectively in the promoter region of *zrfA* and *zrfC*). Besides, in the P*zrfB* fragments, two different ZafA-protected regions were found, that were called ZRB1-2 (for Zinc-Response motif located in the promoter region of *zrfB*) (Figure 5 and Figure S2). It is interesting to recall that in nearly all cases, highly hypersensitive regions to DNase I digestion were observed, that contrasted clearly with the adjacent ZafA-protected regions (Figure S2). The comparison of all ZafA-protected regions brought the ZR consensus sequence 5′-DYYVYCARGGTVYYY-3′ (D = A or G or T; V = A or G or C; R = A or G) (Figure 5), which strongly resembled the M1 motif detected after MEME analysis (Figure 3).

### 3.5. The Highly Conserved 5′-CARGGT-3′ Core of the ZR Motif Is Essential for ZafA Binding

**Figure 6:**
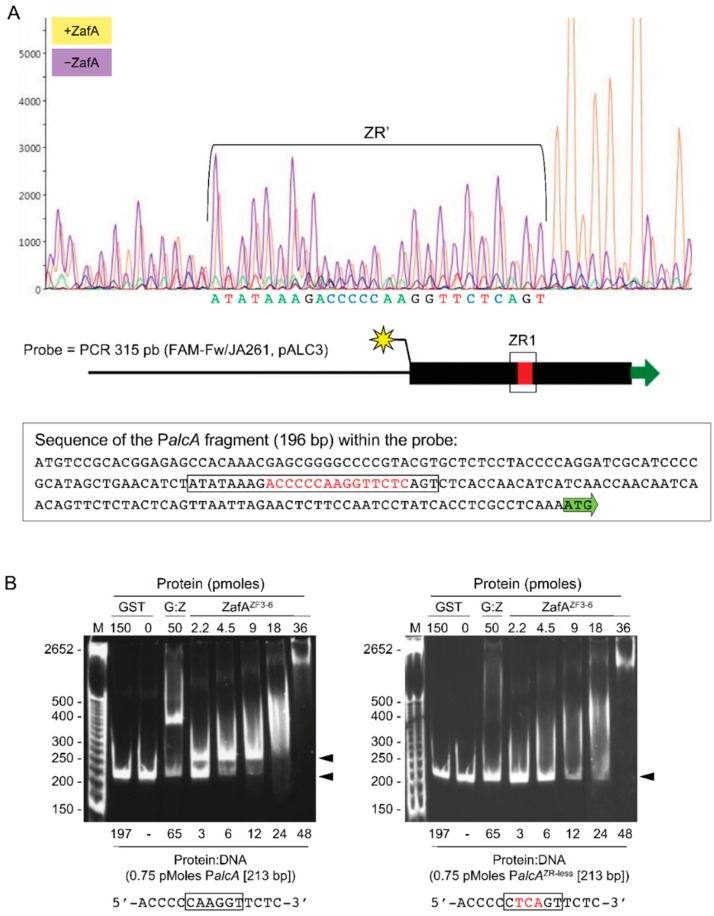
Effect of mutations in the conserved hexanucleotide core of the ZR consensus motif on ZafA binding in vitro. (**A**) Binding of ZafA^ZF3-6^ to a DNA fragment carrying a single ZR consensus motif in a CT-rich context as shown by a DNase I protection assay. The purple chromatogram was obtained upon incubating the DNA fragment in the absence of ZafA^ZF3-6^, such that the DNA remained naked and, hence, unprotected from DNase I digestion. The yellow chromatogram was obtained upon incubating the DNA in the presence of ZafA^ZF3-6^. ZafA bound to DNA prevented its digestion with DNase I resulting in a stretch of yellow peaks lower in high than purple peaks. A short stretch of high yellow peaks at the right side of the chromatogram indicated a hypersensitive region to DNase I digestion. The protected region is delimited by a bracket. Below the chromatogram is shown a schematic representation of the whole intergenic region of the *alcA* gene with the beginning of the ORF located at the right side (green arrow). The sequence of the DNA fragment corresponding to the P*alcA* used in the assay is located in the scheme as a black rectangle with the ZR consensus motif in red. The protected region has been squared in the scheme and sequence. The yellow star indicates the end 6-FAM labeled strand detected by the capillary-based automated DNA sequencer; (**B**) Analysis by EMSA of binding of ZafA^ZF3-6^ to a DNA fragment in which the 5′-CAAGGT-3′ hexanucleotide core of its only ZR motif (left panel) had been mutated to 5′-CTCAGT-3′ (right panel). The DNA fragments were also incubated without protein and in the presence of both 150 pmoles of purified GST and 50 pmoles of the fusion protein GST-ZafA^ZF3-6^ (as controls) to attain a GST:DNA ratio of 197 and a GST-ZafA^ZF3-6^:DNA (G:Z) ratio 65. A 25-bp DNA ladder was used as a reference.

The ZR consensus motif showed a highly conserved 5′-CARGGT-3′ hexanucleotide core that could be essential for ZafA to recognize and bind specifically to it, such that the replacement of a purine by a pyrimidine, or vice-versa, could reduce or impair the binding of ZafA. We thought that this hexanucleotide sequence, rather than the short CT-rich flanking sequences, was required for the specific binding of ZafA. To confirm this, we searched in the *Aspergillus* genome database for a DNA fragment corresponding to the promoter region of any gene with a relatively high number of short CT-rich sequences (≥3 bp) and that contained just one ZR consensus motif, to be used as probe for both EMSA and DNase I footprinting assays. Thus, we selected a 191-bp DNA fragment, the *alcA* gene promoter from *A. nidulans* that met our requirements to be used as a probe. We confirmed by a DNase I footprinting assay that ZafA only protected a region of this DNA fragment that contained the ZR consensus motif (Figure 6A). To ascertain whether the 5′-CARGGT-3′ sequence was essential for ZafA binding, we created a mutated version of this probe in which its 5′-CAAGGT-3′ core had been changed to 5′-CTCAGT-3′. The binding of ZafA to the wild-type P*alcA* probe at ZafA:DNA ratios lower than 6.0 resulted in a retarded DNA:protein complex as shown by EMSA (Figure 6B). In contrast, ZafA did not bind to the P*alcA* probe in which the ZR motif had been mutated. These results showed that ZafA was able to bind to the ZR motif and that the 5′-CARGGT-3′ hexanucleotide core sequence was essential for ZafA to recognize such motif.

### 3.6. *ZafA* Induces Gene Expression in vivo through Binding to the ZR Motifs

**Figure 7:**
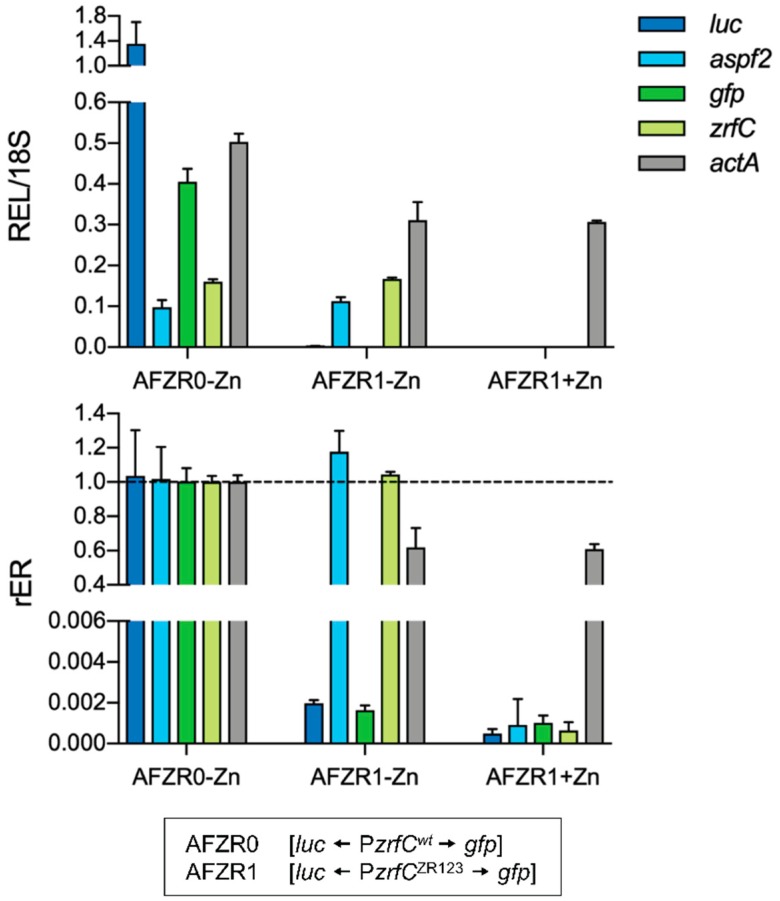
Effect of mutations in the conserved hexanucleotide core of the ZR consensus motif on ZafA binding in vivo. (Upper panel) Relative expression levels (REL) of endogenous *aspf2* and *zrfC* genes and their corresponding reporter genes (*luc* and *gfp*) driven by a wild-type (in the AFZR0 strain) and mutant version (in the AFZR1 strain) or the divergent *aspf2-zrfC* promoter (abbreviated as P*zrfC*). In the mutant version of this promoter the three ZR motifs present in it (P*zrfC*^ZR123^) had been inactivated by site-directed mutagenesis. Both strains were cultured under zinc-limiting conditions in SDN–Zn for 20 h at 37 °C. The AFZR1 strain was also grown under zinc-replete conditions in SDN + 100 µM Zn to be used as a control of the effect of zinc on the transcriptional activity of ZafA on P*zrfC*^wt^. (Lower panel) Relative expression ratio (rER) of data represented in the upper panel after their normalization with respect to the expression level in the AFZR0 strain grown under zinc-limiting conditions. The expression level of the actin gene was also measured by RT-qPCR as an additional control. The 18S rRNA was used as an internal reference for all relative quantifications.

Although mutations within the 5′-CARGGT-3′ hexanucleotide core prevented the binding of the recombinant ZafA^ZF3-6^ protein to the ZR motifs in vitro, it could be possible that in vivo the wild-type ZafA protein binds to a DNA sequence different from the ZR motif determined in vitro. Hence, to ascertain whether ZafA truly bound to the ZR motifs in the fungus growing in zinc-limiting media, we analyzed in vivo the effect of the inactivation of the three ZR motifs (ZR1, ZR2 and ZR3) present in the divergent promoter that drives the transcription of both *aspf2* and *zrfC* (P*zrfC*) on the transcription of these genes [11]. To test this, we introduced, at the *pyrG* locus of a uridine-uracil-auxotrophic strain (CEA17), the coding sequences of both the luciferase (*luc*) and green fluorescent protein (*gfp*) arranged divergently and separated by a P*zrfC* mutant version (P*zrfC*^ZR123^) whose three ZR motifs had been inactivated by replacing each 5′-CAAGGT-3′ hexanucleotide core by the mutated 5′-ACATGT-3′ sequence (Figure S1). It must be noted that, in this construction, the ORF of *aspf2* and *zrfC* had been replaced respectively by that of *luc* and *gfp*. Hence, this construction was devised in such a way that the transcription level of *luc* and *gfp* would indicate the functionality of the mutant promoter (P*zrfC*^ZR123^) in a wild-type genetic background, while the expression levels of the endogenous *aspf2* and *zrfC* genes could be used as internal controls of the transcriptional promoter activity of P*zrfC*^wt^. We expected that if these ZR motifs were truly recognized by ZafA in vivo, the mutation of their conserved hexanucleotide cores should prevent the binding of ZafA to P*zrfC*^ZR^ and the expression of both *luc* and *gfp*. As shown, the transcription of both reporter genes driven by P*zrfC*^ZR123^ in the AFZR1 strain grown under zinc-limiting conditions collapsed as if the fungus was growing in zinc-replete conditions (Figure 7). More precisely, the relative expression ratio (rER) of the *luc* and *gfp* transcripts in AFZR1 was reduced respectively by 523- and 611-fold as measured by RT-qPCR, whereas the relative expression ratios (rERs) of *asfp2* and *zrfC*, which were used as endogenous controls, were identical to that in the control AFZR0 strain that carried the P*zrfC*^wt^. These results undoubtedly showed that ZafA has to bind to the ZR motifs in vivo to induce gene expression under physiological conditions of zinc starvation.

### 3.7. The ZR Motifs Appear to Function as Transcriptional Regulatory Sequences When Located within 1.2 Kb Upstream from the Predicted Translation Start Codon of the ORFs Whose Expression Is Influenced by Zinc

Although the highly conserved 5′-CARGGT-3′ core of the ZR motif was essential for ZafA binding, it exhibited a relatively high degree of variability in the 5′ and 3′-end sequences that flanked the conserved core. This fact could influence the affinity of ZafA binding to the different ZR motifs in the DNA. In addition, it would not be unexpected that ZafA was able to bind also to ZR motifs harboring a certain degree of sequence degeneration. Thus, it could be possible that microarray data under the tested culture conditions had not revealed all ZafA potential targets if the expression of an unknown number of genes were influenced, in addition to zinc starvation, by other environmental signals. Hence, to obtain further evidence on the role of the ZR motif as a regulatory sequence of gene expression under zinc-limiting conditions, we performed a genome-scale search for ZR motifs in the *A. fumigatus* genome by utilizing the RSAT web server [28]. To perform this search, we used as a query pattern the ZR consensus sequence or ZR0 motif, while admitting one substitution out of the conserved hexanucleotide core. Hence, nine additional different degenerated ZR-like motifs (ZR1-9) were possible (Table S8). Since there is one gene every 2938 bp in the genome of *A. fumigatus* and the mean gene length is 1431 bp [30], it can be deducted that the average intergenic sequence is 1482 bp. In order to identify most of the genes harboring ZR-like motifs in their promoter regions, the search for them was extended up to −3000 bp upstream of the predicted translation start codon (TSC) of the open reading frames (ORFs) (i.e., about 2× the size of an average intergenic sequence). By enabling and disabling the overlapping with upstream coding sequences we differentiated two sets of ZR motifs: (1) Non-overlapping ZRs, which were located in the intergenic sequences corresponding to the promoter regions of genes regulated presumably by ZafA; and (2) Overlapping ZRs, which were located either within an upstream ORF or in an intergenic sequence that would correspond to the promoter region of an upstream gene.

A detailed analysis of the non-overlapped ZRs at a genome-scale indicated that there were 545 different ZR motifs distributed among 499 different intergenic sequences harboring the promoter regions that drive the expression of 671 different coding sequences (since 51.3% of such coding sequences were arranged as divergent pairs of genes in the genome). About 91.4% of all genes, including both non-divergent and divergent arranged genes, had in their upstream intergenic regions only one ZR motif, 7.4% had 2 ZRs and 1.2% showed ≥3 ZRs. The most abundant ZR motifs in promoter regions were ZR0, ZR7 and ZR9 (103.3 ± 3.2 per ZR motif). The next most abundant ZR motifs in the promoter regions were ZR2, ZR3, ZR5 and ZR8 (83.5 ± 6.9 per ZR motif). The less abundant ZR motifs were ZR1, ZR4 and ZR6 (31.7 ± 6.7 per ZR motif), as would be expected for the ZR motifs with the lowest variability. Upon analyzing the distribution in the intergenic regions of the most abundant ZRs depending on both the type of ZR and their distance to their correspondent ORFs, it was determined that the overall distribution of the most abundant ZR motifs was influenced very significantly by their distance to the TSC of the ORFs (2-way ANOVA, *p* < 0.0001) but not by the type of ZR motif (2-way ANOVA, *p* = 0.322). Similarly, the overall distribution in the intergenic regions of the less abundant ZR motifs (ZR1, ZR4 and ZR6) was also influenced significantly by their distance to the TSC of the ORFs (2-way ANOVA, *p* = 0.0021) but not by the type of ZR motif (2-way ANOVA, *p* = 0.36). Taken together, these findings suggested that the distance of the ZR motifs to the TSC of the ORFs appeared to be an important factor that determined their distribution in the intergenic promoter regions.

**Figure 8:**
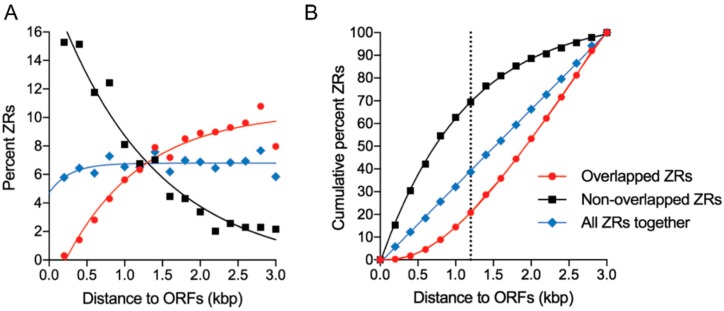
Distribution of ZR motifs at a genome scale in the upstream regions of the predicted open reading frames (ORFs). (**A**) Representation of the percentage of ZR motifs (overlapped, non-overlapped and all together) with respect to their distance to the predicted translation start codons (TSC) of the ORFs; (**B**) representation of the cumulative percent of ZR motifs with respect to their distance to the predicted translation start codons of the ORFs. The dotted line indicates the average size of the DNA sequence that is located upstream of the predicted TSCs containing the ZR motifs with the highest probability of being involved in regulating gene expression (i.e., the functionally relevant ZR motifs). In both graphs, the distance of the ZR motifs to the ORFs represented on the x-axes was divided into 15 fractions of 200 bp each.

We then analyzed the distribution of all ZR motifs (i.e., non-overlapped plus overlapped ZRs) with respect to their distance to the ORFs. The results revealed that, as expected for a random distribution of ZRs along the DNA, the percentage of ZR motifs within each 200 bp DNA fraction, in which was arbitrarily divided the DNA sequences of 3000 bp located upstream the ORFs, maintained constant between 5.8–7.7% (Figure 8A; blue symbols). Accordingly, the cumulative percent of all ZR motifs (i.e., including both non-overlapped and overlapped ZR motifs) increased directly proportional to the distance to the ORFs (R^2^ = 0.999), as would be expected for a random distribution (Figure 8B; blue symbols). However, when the distribution of both non-overlapped and overlapped ZR motifs were analyzed separately, they showed opposite distribution patterns. Thus, within each 200 bp DNA fraction, the percent of non-overlapped ZR motifs (Figure 8A; black symbols) reduced exponentially (R^2^ = 0.963) from 15.3% to 2.2%, whereas that of the overlapped ZR motifs (Figure 8A; red symbols) increased exponentially (R^2^ = 0.955) as their distance to the ORFs increased from 0.3% to 11.0%. Accordingly, the increase of the cumulative percent of non-overlapped ZR motifs reduced gradually with the distance to the ORFs at a rate that was proportional to the amount of the remaining ZR motifs, i.e., according to an exponential one phase decay relationship (R^2^ = 0.999) (Figure 8B; black symbols), as would be expected if the ZR motifs were required to be located in a relatively close proximity to the ORFs for them to function as regulatory motifs. In contrast, within the analyzed range (i.e., between −1 to −3000 bp upstream of the ORFs) the increase in the cumulative percent of the overlapped ZR motifs increased with the distance to the ORFs according to a third order polynomial relationship (R^2^ = 0.999) (Figure 8B; red symbols). Interestingly, the graphical representation of this function showed two phases: An initial exponential increase of the cumulative percent of the overlapped ZR motifs (until is reached 1200 bp upstream the ORFs) followed by an increase in the cumulative percent of overlapped ZR motifs that was directly proportional to the distance to the ORFs. The exponential part of this representation reflected the distribution of regulatory ZRs located in the intergenic promoter sequences of upstream genes with very short ORFs that were located in the near vicinity of the reference gene; whereas the arithmetic part of the representation reflected the random distribution of the ZR motifs 1200 bp upstream the TSCs. Besides, this distance matched perfectly the average size of the upstream distance to ORFs that was defined by the highest difference between the cumulative percent of overlapped and non-overlapped ZRs located within the 3000 bp upstream the ORFs (Figure 8B).

Interestingly, the same tendency was observed for pairs of genes arranged divergently whose intergenic regions contained ZR motifs. Thus, the gene having most of the ZR motifs located at less than 1200 bp from the predicted TSC had a higher probability of being regulated by ZafA than the gene that had the ZR motifs at a distance >1200 bp of its TSC. For instance, in the intergenic region of the divergent pair of genes AFUA_1G01560/AFUA_1G01550 there were three motifs located respectively at −75, −105 and −157 bp from the TSC of AFUA_1G01550 (*zrfA*) and at −1784, −1754 and −1702 bp from the TSC of AFUA_1G01560. However, only the AFUA_1G01550 gene (*zrfA*) was induced by ZafA. In contrast, those genes arranged divergently and separated by intergenic sequences < 1200 bp are co-regulated simultaneously by ZafA (e.g., gene pairs AFUA_4G09560/AFUA_4G09580, AFUA_4G03920/AFUA_4G03930 or AFUA_8G02450/AFUA_8G02460). These results suggest that the ZR motifs located within 1200 bp upstream of the TSC of an ORF appear to be functionally relevant in the ZafA-mediated regulation of gene expression under zinc-limiting conditions.

Finally, a comparison of genes detected by microarrays in a wild-type strain as differentially regulated under zinc starvation with that found in silico harboring, non-overlapping ZR motifs in their promoter regions revealed that 31 DZR genes (i.e., 20.3% of the 153 DZR genes) and 55 non-DZR genes (i.e., 8.6% of 641 non-DZR genes) had ZR motifs in their promoters. Interestingly, 150 SSA genes (i.e., 7.0% of 2130 SSA genes) also had non-overlapping ZR motifs in their promoter regions. In addition, 30 DZR genes had their ZR motifs located within 1200 bp from their predicted TSC (i.e., 96.8% of all DZR genes with ZRs). In contrast, the weighted average percent of the non-DZR genes with ZR motifs located within 1200 bp from their predicted TSC was 71.2%. Taken together, these results indicated that ZR motifs predicted to be functionally relevant were located in the promoters of 19.6% of the DZR genes but only in 5.4% of genes not regulated directly by ZafA.

### 3.8. Putative Role of *ZafA* as a Repressor under Zinc-Limiting Conditions and Identification of the Most Likely Direct Target Genes of *ZafA*

The data obtained from the microarray experiments showed that, of all genes from a wild-type strain of *A. fumigatus* whose transcription level was influenced under zinc-limiting conditions, 64.5% were down-regulated whereas 35.5% were up-regulated (Figure 2). In contrast, 22.9% of the DZR genes were down-regulated whereas 77.1% were up-regulated. These results suggested that ZafA functioned mainly as a transcriptional activator rather than as a repressor.

In the microarray version used in this study, the AFUA_7G06570 gene (*zrcA*) had not been included. However, this gene encodes an important putative vacuolar zinc transporter of the CDF family likely involved in the homeostatic response to zinc excess [10]. Besides, it harbors one copy of the ZR consensus motif in its promoter region. Thus, we investigated whether the *zrcA* transcription was also influenced by ZafA under zinc-limiting conditions. It was shown by RT-qPCR that the expression level of *zrcA* reduced by about 6-fold in a wild-type strain grown in a zinc-limiting versus a zinc-replete medium, while it increased by 16-fold in a ∆*zafA* mutant grown in a zinc-limiting medium compared to a wild-type strain grown in the same conditions, i.e., ZafA was formally a repressor of *zrcA* under zinc-limiting conditions (Table 2). Hence, it would be theoretically possible that ZafA also down-regulated the expression of other DZR genes. In this regard, we confirmed by RT-qPCR that ZafA could also be formally considered as a repressor of the genes AFUA_2G01260/*srbA* [ZR3], AFUA_2G15290 [ZR5], AFUA_5G06240/*alcC* [ZR8] and AFUA_6G00690 [ZR5] under zinc-limiting conditions (Table 2). Importantly, all of these genes carried ZR motifs with one substitution in their promoter regions.

On the other hand, it was shown previously through a combination of in vitro and in silico analyses that at least 30 DZR genes with ZR motifs located within 1200 bp from their predicted TSC were putative direct ZafA targets. However, we also observed under zinc-limiting conditions, that some induced (e.g., *gtmA*, *pyroA*, *azf1*, *gliA*, *gliT*) and repressed genes (e.g., *sod1*, *erg24A*) (Table 2), which according to their expression profiles should be formally considered as direct ZafA targets, did not have obvious ZR motifs in their promoter sequences. However, after a search for non-overlapping ZR motifs with two substitutions in the promoter regions (until 1200 bp upstream of the predicted TSCs) using the RSAT software and the ZR consensus motif (ZR0) as a query sequence, 18 additional DZR genes harboring ZR degenerated motifs were found. Interestingly, among these genes were AFUA_6G09310 and AFUA_5G09240/*sodA*, which were ZafA-repressed genes, and *gliA*, which was one of the most strongly induced genes under zinc-limiting conditions. More importantly, this research also allowed us to detect additional ZR motifs in the promoter regions of genes encoding both important zinc homeostatic proteins (e.g., one additional site was detected in *zrfA* and *zafA* thus bringing the total number of ZRs in the promoters of these genes to 3 and 4 respectively) and other genes of unknown function but whose expression was also strongly induced by ZafA under zinc-limiting conditions (e.g., one additional ZR motif was detected in the AFUA_7G06810/*sarA* promoter and the AFUA_8G02460-AFUA_8G02450/*gzbA* divergent promoter; two additional ZR motifs were detected in the AFUA_8G02620/*mchC* promoter and the AFUA_4G03920-AFUA_4G03930/*cysX* divergent promoter).

**Figure 9:**
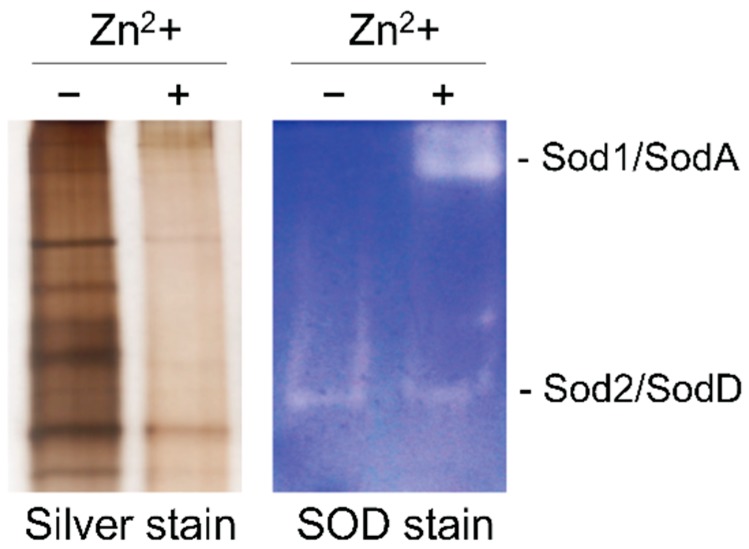
Detection of superoxide activity onto PAGE gels. Total extracts of native proteins were obtained from a wild-type strain of *A. fumigatus* grown under zinc-limiting (SDN–Zn) and zinc-replete conditions (SDN + 100 µM Zn), separated in a PAGE gel (T = 8%) and stained as described in the materials and methods section. Sod1/SodA is a cytosolic Cu/Zn-dependent superoxide dismutase whereas Sod2/SodD is a mitochondrial Mn-dependent superoxide dismutase.

Finally, it is interesting to recall that unlike the expression level of genes encoding zinc-independent enzymes typically involved in defense against reactive oxygen species (ROS), such as *cat1*, *cat2* and *sodC*; increases under zinc-limiting conditions, the expression level of *sodA*, which encodes a cytosolic Zn/Cu-superoxide dismutase, is reduced most likely as a mechanism to reduce the biosynthesis of the enzyme, since its functionality is strongly reduced under zinc-limiting conditions (Figure 9).

In summary, these results supported the notion that the ZR consensus motif and other ZR motifs with different degeneration degrees out of the 5′-CARGGT-3′ hexanucleotide core, could function as regulatory sequences to which ZafA would bind under zinc-limiting conditions, and be brought to 49, the total number of DZR genes with ZR motifs (i.e., 31.8% of DZR genes) whose expression was regulated directly by ZafA, including 20 confirmed and 29 predicted genes (Table 3).

## 4. Discussion

The effect of zinc-deficiency on the regulation of gene expression and the homeostatic and adaptive response to zinc starvation has been extensively analyzed at a genome-wide scale in the yeast *Saccharomyces cerevisiae* [16,17,18]. In this yeast, the regulatory response to zinc starvation at the transcriptional level relies on the Zap1 transcription factor [31]. We described for the first time that regulation of zinc homeostasis in a fungal pathogen is essential for virulence [13,14]. Similar studies, conducted later in different *Candida* and *Cryptococcus* species, *Histoplasma capsulatum* and *Blastomyces dermatitidis*, have also highlighted the importance of maintaining zinc homeostasis for the virulence of these pathogenic yeast [19,20,32,33,34,35,36]. In this paper, we report the transcriptional profiling of *A. fumigatus* under zinc starvation and show that ZafA regulates the expression of its most direct target genes through binding to one or more ZR consensus motifs located in their promoter regions.

In both *S. cerevisiae* and the pathogenic fungi studied thus far, the genes more strongly induced by the ZafA/Zap1 orthologues encode membrane transporters of the ZIP family, involved in zinc uptake from zinc-limiting media. Besides, it has been observed that zinc-deficiency increases ROS production [20,33,37] while it reduces sulfate assimilation as a putative mechanism to alleviate oxidative stress under zinc-limiting conditions [38]. In this regard, the adaptive response to zinc deficiency is well mirrored at the transcriptional level by changes in the expression of genes encoding proteins involved in ROS detoxification and assimilation of inorganic sulfur both in *S. cerevisiae* [18] and in all pathogenic yeasts [19,20]. Similarly, in *A. fumigatus* grown under zinc-limiting conditions, genes encoding zinc-independent proteins involved in ROS detoxification (e.g., *cat1*, *cat2* and *sodC*) are up-regulated, while *metR* [39], which is the major regulator of inorganic sulfur assimilation, is down-regulated (Table 2 and Table S6). There are, however, some remarkable differences between species regarding specific aspects. Among the most noteworthy are those related to iron metabolism, ergosterol biosynthesis and protection against oxidative stress, which appears to be linked to the production of certain secondary metabolites.

The major difference between *A. fumigatus* and the yeasts *S. cerevisiae*, *C. albicans* and *C. gatti* regarding iron metabolism is that only *A. fumigatus* is able to synthesize and secrete siderophores for iron uptake under iron-limiting conditions [40]. The only gene related to iron homeostasis that has been reported to be induced by Zap1 in *S. cerevisiae* under zinc-limiting conditions is *FET4* [16,18], which encodes a low-affinity Zn^2+^/Fe^2+^/Cu^+^ transporter [41]. In *C. albicans* grown under zinc-limiting conditions, the expression of some genes encoding proteins of the reductive pathway (e.g., *FRE2* and *FET34*) is up-regulated, whereas the expression of others is down-regulated (e.g., *FRE5*, *FRE7*, *FRE10*, *FET3* and *FTH1*). In *C. gatti* grown under zinc-limiting conditions, the expression of the genes CNBG_3602 and CNBG_2036 is down-regulated [20]. They encode proteins similar to FtrA and MirB of *A. fumigatus* that function respectively in the reductive and non-reductive/siderophore-mediated iron uptake system. In *A. fumigatus* are down-regulated, under zinc-limiting conditions, several genes encoding proteins that are involved in iron uptake, including the FetC/FrtA oxidase/permease complex of the reductive iron uptake system and several non-reductive siderophore transporters of *A. fumigatus* (Table 2 and Table S6). In addition, genes encoding regulators of iron homeostasis (e.g., *hapX* and *srbA*) and siderophore biosynthesis are also down-regulated in *A. fumigatus* under zinc starvation (Table 2 and Table S6). Therefore, provided that all transcriptional profiling studies performed thus far in fungal pathogens have been done under non-iron-limiting conditions, it is concluded that the expression of genes encoding proteins required for iron uptake appear to be reduced under zinc-limiting conditions, which indicates that a reduction of iron intake from non-iron-limiting media appears to be an adaptive response to zinc deficiency. Whether Zap1 plays a direct or indirect role in regulating the expression of genes involved in iron homeostasis in yeast has not been reported [19]. However, it appears that in *A. fumigatus* growing under zinc-limiting conditions, only the expression of some genes related to iron homeostasis is directly or indirectly regulated by ZafA (e.g., *srbA*, *enb1*, *mirB* and *sidA*), whereas most of them appear to be regulated in a ZafA-independent manner (Table 2 and Table S6). On the other hand, the transcriptional profiling of *A. fumigatus* grown in zinc-replete media following a shift from iron-limiting to iron-replete conditions has revealed that the expression of genes typically involved in the homeostatic response to zinc deficiency (e.g., *zafA*, *zrfA* and *zrfB*) is up-regulated whereas that of genes involved in the homeostatic response to zinc excess is down-regulated (e.g., *zrcA*) [42]. The expression profile of these ZafA-regulated genes in the referred conditions has been interpreted as a homeostatic mechanism to prevent zinc excess and zinc toxicity under iron starvation [43]. In addition, in a wild-type strain growing in zinc-replete media under iron-replete conditions HapX mediates both the up-regulation of genes typically involved in the homeostatic response to zinc deficiency (e.g., *zafA* and *zrfB*) and the down-regulation of genes involved in the homeostatic response to zinc excess (e.g., *zrcA* and *zrcC*) [44]. However, it is known that the expression of *hapX* is reduced dramatically under iron-replete conditions [44], which indicates that the predicted action of HapX on the expression of the aforementioned genes must be exerted by the low basal amount of HapX that is synthesized under iron-replete conditions [45]. It is difficult to understand that the HapX-mediated increase of the transcription level of *zafA*, which encodes the key major regulator of zinc homeostasis, only reflects changes in the expression level of *zrfB* and *zrcA* (of all putative direct targets of ZafA). Nevertheless, this could be explained upon considering that *zrfB* is the zinc homeostatic gene with the highest number of ZR motifs in its promoter, whereas *zrcA* is one of the few genes whose expression is strongly repressed by ZafA that harbors one ZR consensus motif in its promoter region (Table 3). Hence, it could be possible that the low increase in the expression level of *zafA* detected in a wild-type strain versus a ∆*hapX* strain (about 1.8-fold) [44], allows synthesizing a little (although sufficient) amount of ZafA. This regulator could bind preferentially to the promoters of *zrfB* and *zrcA* to induce the former and repress the later. The *zrcC* gene does not have ZR motifs in its promoter region and most likely its repression under zinc-limiting conditions is not directly mediated by ZafA. The HapX-mediated co-regulation of the *zrfB, zrcA* and *zrcC* expression in *A. fumigatus* might have evolved as an adaptive response to iron excess caused by a sudden iron supplement under zinc-replete conditions. This response would prevent the rapid storage into the vacuole (mediated by ZrcA and ZrcC) of the zinc taken up by ZrfB. The most likely reason would be that a concentration of cytosolic zinc higher than usual is required to prevent iron toxicity during a transient iron excess. This adaptive response recalls the proactive mechanism that operates in *S. cerevisiae* to protect cells against a sudden high increase of zinc concentration in the medium or “zinc shock” [46]. This mechanism relies on the function of *ZRC1* that is the orthologue of the *zrcA* gene of *A. fumigatus*. *ZRC1* is up-regulated by Zap1 to enhance the storage into the vacuole of zinc that is incorporated by Zrt1 under zinc-limiting conditions [46,47]. Provided that these studies have been performed in media containing a relatively low amount of iron such as that present in the yeast nitrogen base (i.e., 0.74 µM) used to make the yeast synthetic minimum medium, it could be possible that zinc storage following a “zinc shock” is intended to prevent zinc toxicity due to iron insufficiency. In summary, a mutual relationship between regulation of homeostasis of zinc and iron appears to exist. This zinc/iron cross-homeostasis could be essential for fungal growth in zinc-replete media. It would not only prevent the excessive zinc uptake and zinc toxicity under iron starvation, as proposed previously [43], but also it might increase the cytosolic concentration of zinc to prevent iron toxicity under iron-replete conditions and/or prevent an excessive iron uptake from non-iron-limiting media and iron toxicity under zinc starvation. Consequently, the zinc/iron cross-homeostasis would allow the fungus to grow well in media containing unbalanced Zn:Fe ratios.

Some NZR genes are down-regulated, both in zinc-limiting media under iron-replete conditions and in zinc-replete media after a shift from iron-limiting to iron-replete conditions, such as *hmg1*, which encodes the enzyme of the committed step in ergosterol biosynthesis, and several genes related to siderophore biosynthesis (e.g., *sidC*, *sidF*, *sidG*, *amcA*) [44]. This finding suggests that the reduction of both iron uptake and ergosterol biosynthesis might also have co-evolved as part of the fungal adaptive response both to zinc starvation under non-iron-limiting conditions, and to iron excess under non-zinc-limiting conditions. In addition, a relatively high number of genes whose expression is regulated by the sterol-response element binding proteins (SREPBs), SrbA and SrbB, are also influenced by zinc starvation [48,49]. For instance, several genes involved in ergosterol biosynthesis (i.e., *erg1*, *erg3A*/*B*, *erg5*, *erg13B*, *erg24A*/*B*, *erg25A*/*B* and *hmg1*), ethanol catabolism (*alcC*), heme biosynthesis (*hem13* and *hem14*) and nitrate assimilation (*niiA* and *niaD*), are up-regulated by SREPBs in hypoxia and down-regulated under (normoxic) zinc-limiting conditions (Table 2). Similarly, the own expression of *srbA* and *srbB* is strongly reduced under (normoxic) zinc-limiting conditions (Table 2). On the other hand, it is known that the transcription of *srbB* is autoinduced by SrbB and starts earlier (and is stronger) than that of *srbA*, which is autoinduced by SrbA [49]. Besides, the RNAseq data for the wild-type, Δ*srbA* and Δ*srbB* strains, after 2 h in hypoxia, revealed that the expression level of *srbB* in the Δ*srbA* mutant is lower than in the wild-type strain; whereas the expression level of *srbA* in the Δ*srbB* mutant is higher than in the wild-type strain, i.e., SrbA is formally an inductor of *srbB* whereas SrbB is a repressor of *srbA* [49]. Since *srbA* can be formally considered as a direct ZafA target (Table 2), it could be possible that *srbA* repression initiated by ZafA, under normoxic zinc-limiting conditions, triggers a cascade of transcriptional events that ultimately results in the strong down-regulation of *srbA* and *srbB* (and that of their target genes) under sustained zinc-limiting conditions. However, the same RNAseq experiment also indicated that SrbA and SrbB are formally positive regulators of *zrfA*, *zrfB* and *zrfC* but negative regulators of *zafA* [49]. In addition, SrbA is a positive regulator of *zrcA* and *zrcC* whereas SrbB is a negative regulator of these genes [49]. Hence, provided that these studies have been performed in zinc-replete media, the positive regulation of *zrfA*, *zrfB* and *zrfC* by SrbA and SrbB, along with the early negative regulation of *zrcA* and *zrcC* by SrbB, this suggests that a cytoplasmic zinc concentration higher than usual may be beneficial for fungal growth in hypoxia. Of note, this regulatory mechanism for the adaptive response to hypoxia resembles the one involved in preventing iron toxicity during iron excess under zinc-replete conditions. In addition, it has been reported that the fungal zinc content is about 3-fold higher in mycelium grown in hypoxia than in normoxia [50]. However, the SREPB-mediated down-regulation of *zafA* by SrbA and SrbB under hypoxic zinc-replete conditions suggests that the expression *zrfA*, *zrfB* and *zrfC* should be induced directly by these factors instead of being induced by ZafA*.*

*S. cerevisiae* and *C. albicans* lack SREPBs orthologues and appear to have evolved different regulatory mechanism [51]. In either case, under hypoxic (zinc-replete) conditions the expression of the *ERG* genes is down-regulated in *S. cerevisiae* [52], but up-regulated in *C. albicans* [53] and *A. fumigatus* [48]. This suggests that the sterol content in *S. cerevisiae* under hypoxic conditions is largely sufficient to sustain growth whereas in *C. albicans* and *A. fumigatus* it is largely insufficient, thus requiring the increase of the expression of the ergosterol biosynthetic genes encoding enzymes that catalyze oxygen-dependent reactions (Erg1, Erg11, Erg25 and Erg3). However, it has been reported that the expression of the *ERG* genes is down-regulated in a *S. cerevisiae* Δ*zap1* mutant [16], which indicates the ScZap1 is formally a positive regulator of the *ERG* genes. In contrast, in a *C. albicans* Δ*zap1* mutant the expression of the *ERG* genes is up-regulated [19], which indicates the CaZap1 is formally a negative regulator of the *ERG* genes. However, the *ERG* genes in *S. cereviasie* and *C. albicans* lack Zap1 binding motifs in their promoter regions, which suggests that both ScZap1 and CaZap1 regulate indirectly the expression of these genes [19]. Similarly, the *erg* genes of *A. fumigatus* lack ZR motifs in their promoters and, under zinc-limiting conditions, only a few *erg* genes (e.g., *erg5*, *erg13B*, *erg24A*, *erg24B* and *erg3B*) are likely repressed either directly or indirectly by ZafA, whereas it is likely that the expression of other genes (e.g., *hmg1*, *erg1*, *erg3A*, *erg25A* and *erg25B*) are down-regulated in a ZafA-independent manner (Table 2 and Table S6). In either case, one possibility is that the repression of the *erg* genes under normoxic zinc-limiting conditions is an indirect consequence of *srbA* repression by ZafA.

Zinc starvation increases the expression level of genes encoding components of the electron acceptor complexes I-III of the mitochondrial electron transport chain (ETC) (e.g., *sdh3* and *sdh4* that encode the inner-membrane-anchored subunits of the succinate deshydrogenase), while it reduces the encoding of the mitochondrial fumarate reductase (FrdA) and some genes encoding components of complex IV of the ETC (e.g., AFUA_2G03010 and AFUA_3G14440). The *frdA* gene, which is one of the most strongly induced genes during hypoxia in *A. fumigatus* [54], encodes a FAD-dependent enzyme similar to the yeast Frd1 enzyme that couples the reduction [fumarate → succinate] to the oxidation [ubiquinol → ubiquinone] (i.e., the reverse reaction that is catalyzed by the succinate deshydrogenase) [55]. The complexes I and III are considered as major sites of ROS generation in mitochondria [56]. Thus, it is tempting to speculate that the imbalance between the expression of genes encoding components of the electron acceptor complexes I/II and electron donor complex IV under zinc-limiting conditions may disturb the normal electron flux through the ETC in normoxia. In this scenario, not all electrons that enter the ETC are finally used for O_2_ reduction, at the same time that the intracellular partial pressure of O_2_ in the fungus growing under zinc-limiting conditions becomes relatively higher than in the fungus growing in zinc-replete media. As a consequence, the intracellular O_2_ may reach a slightly hyperbaric concentration while it increases the probability of adventitious reactions of reduced flavins with O_2_ leading to the formation of (ROS) [57]. It is known that enzymes of the succinate/fumarate oxidoreductase family, which include the succinate dehydrogenases and fumarate reductases, are prone to react adventitiously with O_2_ and autoxidation resulting in the formation of superoxide anion and/or H_2_O_2_ (because they contain flavins that appear to be the primary site of electron transfer to O_2_) [57,58]. For instance, the *E. coli* NadB flavoprotein, which is structurally and evolutionary related to succinate/fumarate oxidoreductases, accounts for about 25% of all H_2_O_2_ generated endogenously during aerobic growth [59]. Thus, the reduction of the expression level of *frdA* could be interpreted as a mechanism to prevent an excess of ROS formation under zinc-limiting conditions.

Interestingly, the adaptive response against oxidative stress displayed by *A. fumigatus* appears to differ significantly from that displayed by the yeast *S. cerevisiae*, in which only two gene-encoding enzymes for protection against ROS, *SOD1* and *TSA1*, appear to be critical for growth in low zinc [37,60]. The expression level of *SOD1* in *S. cerevisiae*, which encodes the orthologue to the *A. fumigatus* SodA Cu/Zn-superoxide dismutase, is similar under both zinc-replete and zinc-limiting conditions [60]. In contrast, in *A. fumigatus* the expression level of *sodA* reduced significantly under zinc-limiting conditions, most likely as a mechanism to reduce the biosynthesis of the Cu/Zn-SOD whose functionality is strongly reduced under zinc-limiting conditions (Figure 9). Besides, *A. fumigatus* lacks an obvious orthologous of *TSA1*, which encodes a protein with a dual peroxiredoxin-chaperone function that enables yeast cells to adapt both oxidative stress and deficient folding caused by zinc starvation [37,61]. In summary, the adaptive response against oxidative stress in *A. fumigatus* under zinc-limiting conditions might rely on a dual regulatory mechanism that involves both the down-regulation of genes encoding ROS generating enzymes and the up-regulation of genes encoding ROS detoxifying, zinc-independent enzymes.

Unlike pathogenic yeast, *A. fumigatus* has at least 26 biosynthetic gene clusters (named as BGC1-26) that enables it to produce a great diversity of secondary metabolites and mycotoxins [62]. Interestingly, the expression level of 17 genes belonging to 11 different BGCs are differentially regulated under zinc-limiting conditions (Table S6). The BGC-20 dedicated to gliotoxin (GT) biosynthesis appear to be the most largely influenced by zinc starvation, as revealed by the high expression level of *gliZ*, *gliT*, *gliA* and *gtmA*. GliT catalyzes disulfide formation during GT biosynthesis, whereas GliA is involved in GT efflux and both proteins provide self-protection against exogenous and endogenous GT [63,64,65]. The enzyme GtmA catalyzes the conversion of both the endogenous dithiol-GT (GT-[H_2_S]) and the exogenous GT into bisdethiobis(metylthio)gliotoxin (BmGT), which is a negative modulator of GT biosynthesis, using S-adenosylmethionine (SAM) as a methyl donor [66]. Hence, the transcriptional profile of *gliZ*, *gliT* and *gliA* strongly suggests that either a much higher amount of GT is synthesized in zinc-limiting media than in zinc-replete conditions, or GT is far more noxious for fungal growth in zinc-limiting than in zinc-replete media. Besides, the GtmA-mediated bis-thiomethylation of GT is a SAM-dependent reaction that produces as a by-product S-adenosyl-homocysteine (SAH). SAH is then presumably hydrolyzed to homocysteine (Hcy) by the putative S-adenosyl-homocysteinase encoded by AFUA_1G10130/*sahA*, whose expression is also slightly up-regulated under zinc-limiting conditions (Table 2). It has been proposed that GliT, GliA and GtmA work in concert in *A. fumigatus* to resist the effect of exogenous GT in zinc-replete conditions [67], and that GliT and GtmA are up-synthesized in the presence of both GT and GT plus H_2_O_2_ under zinc-replete conditions [68]. In addition, GT inhibits H_2_O_2_-induced oxidative stress [68,69] because most likely GT is able to replace the function of 2-Cys peroxiredoxins as an electron acceptor in the thioredoxin-peroxiredoxin system [70], which is consistent with the lack of a Tsa1-like protein in *A. fumigatus*. Hence, it is very likely that the concerted action of the GliT, GliA, GtmA and SahA proteins has evolved in *A. fumigatus* as part of the adaptive response against oxidative stress caused by zinc starvation. However, the success of the GT-based strategy to reinforce the ROS detoxification capability of *A. fumigatus* relies on an efficient self-protection mechanism against the toxic effect of an excessive amount of GT. At the same time, a relatively high SAM:SAH ratio must be kept to prevent the competitive inhibition of specific methyltransferases by SAH.

The comparison of genes whose expression levels change in a wild-type strain in zinc-limiting versus zinc-replete conditions with those whose expression levels change in a Δ*zafA* mutant versus a wild-type strain when both are grown under in zinc-limiting conditions, produced a group of genes that was expected to be enriched in the most direct ZafA targets. Thus, we identified 153 putative ZafA target genes (35 down-regulated and 118 up-regulated) that represented about 1.56% of the 9824 predicted ORFs in *A. fumigatus*. Following a similar approach for the characterization of the Zap1 zinc-responsive regulon in *S. cerevisiae,* 111 genes were found to be putatively up-regulated by Zap1 [16], which represent about 1.82% of the 6091 biologically significant ORFs of *S. cerevisiae* [71]. In addition, by using a combination of in vitro and in silico approaches, it was determined that most of the 111 putative Zap1 target genes carried, at their promoter regions, at least one zinc response (ZR) consensus sequence 5′-ACCYYNAAGGT-3′ (or a degenerated version of it) to which Zap1 may bind [18,72]. Thus, it was concluded that a total of 81 genes in the yeast genome were direct targets of Zap1 [18]. Using a similar experimental approach, we have found 49 direct targets of ZafA, including 29 potential and 20 confirmed ZafA targets (Table 3). However, several DZR genes (*erg24A*, *gtmA*, *pyroA*, *azf1*, *gliT* and *frdA*) (Table 2), formally considered as direct targets of ZafA, lack obvious ZR motifs. This suggests that ZafA could be able to bind to ZR motifs with a relatively high degeneration degree. In this regard, it must be aware that, unlike Zap1 of *S. cerevisiae*, which binds to a 11-nucleotide sequence with a relatively low degeneration degree (5′-ACCYYNAAGGT-3′; with 16 [2^2^ × 4] different possible ZR sequences), the ZafA transcription factor of *A. fumigatus* binds to a 15-nucleotide zinc response sequence with a higher degeneration degree (5′-DYYVYCARGGTVYYY-3′; with 3456 [3^3^ × 2^7^] different possible ZR sequences), even without considering that additional substitutions are possible out of the conserved core (5′-CARGGT-3′) of the ZR motif. Unlike this hexanucleotide sequence that is essential for ZafA to bind specifically to the ZR motifs, the high variability of both ends of the ZR motif could be intended to determine the affinity of ZafA binding to the ZRs. In either case, it appears that the diversity of ZR sequences to which ZafA can bind in *A. fumigatus* is noticeably higher than those recognized by Zap1 in *S. cerevisiae*. This fact complicates the identification of functionally relevant ZR motifs in many genes of *A. fumigatus*, including some of the most strongly expressed DZR genes, such as *gtmA* and *gliT*.

It is clear that ZafA plays a role in *A. fumigatus* similar to that of Zap1 in *S. cerevisiae* and in the pathogenic yeast *C. albicans* and *C. gattii*, although the only significant identity among these proteins is restricted to the C-terminal DNA binding domain. However, unlike ScZap1 that has four zinc-fingers for DNA binding (one of the CWCH2-type plus three of the C2H2-type), CgZap1 and CaZap1, similar to ZafA, have five (one of the CWCH2-type plus four of the C2H2-type) (Figure 1). Interestingly, given that a zinc-finger binds typically to a 3-nucleotide sequence [73], it may explain that ZafA binds to a 15-nucleotide ZR motif whereas ScZap1 binds to a 11-nucleotide ZR motif. It is interesting as well to note that the high hypersensitivity of the DNA fragments from the *zafA*, *zrfA*, *zrfB* and *zrfC* promoters to DNase I digestion, observed after performing the in vitro footprinting assays, could be an indication that binding of ZafA to the referred promoters causes DNA bending in vivo as part of the transcriptional regulatory mechanism mediated by ZafA, as reported for other regulators [74].

Finally, it is noticeable that most of the DZR genes that harbored ZR motifs in their promoters are up-regulated, which strongly suggests that ZafA mainly functions as an activator rather than as a repressor. Nevertheless, there are also some down-regulated genes that appear to be directly repressed by ZafA (e.g., *zrcA*, *srbA*, *alcC*, *sodA* and others without obvious ZR motifs in their promoters, such as *frdA* and *erg24A*) (Table 3). Similarly, Zap1 of *S. cerevisiae* appears to function nearly exclusively as a transcriptional activator since all 81 proposed Zap1 target genes appear to be induced by Zap1 [16,18], although it has been reported that Zap1 also functions paradoxically as a repressor of *ZRT2* in low zinc [75]. In addition, Zap1 under zinc deficiency represses the expression of the genes *ADH1* and *ADH3*, which encode zinc-dependent alcohol dehydrogenases, most likely as a mechanism to prevent their synthesis under zinc-limiting conditions [76]. Interestingly, a similar rationale could also be applied to explain the repression by ZafA of zinc-dependent enzymes of *A. fumigatus* such as *alcC* and *sodA*.

## 5. Conclusions

The analysis of the transcriptional profile of the ZafA regulon of *A. fumigatus*, under zinc-limiting conditions, confirmed that ZafA is the major regulator of the zinc homeostatic response to zinc starvation. It also indicated that ZafA, along with other regulators, was involved in a zinc/iron cross-homeostatic controlling network that allows the fungus to grow in media containing unbalanced Zn:Fe ratios. This study suggested as well that reduction of iron uptake and ergosterol biosynthesis might have co-evolved as part of the fungal adaptive response to iron excess under non-zinc-limiting conditions and to zinc starvation under non-iron-limiting conditions, even though this regulation appeared to be exerted only in part and indirectly by ZafA. In agreement with previous reports by other investigators, these results also suggested an increase in ROS production under zinc starvation that *A. fumigatus* counteracted by reducing the expression of genes encoding enzymes involved in ROS production and increasing the expression of genes encoding ROS-detoxifying enzymes and gliotoxin production, along with a self-protection mechanism against endogenous gliotoxin. Finally, it was shown that although ZafA appeared to function mainly as an inductor of gene expression under zinc-limiting conditions, it could also function as a repressor of a limited number of genes. Importantly, ZafA would exert its function as a transcriptional activator through binding to a well-defined zinc response consensus motif (5′-DYYVYCARGGTVYYY-3′) present in the promoters of its most direct target genes. However, ZafA could also bind to ZR motifs with a relatively high degeneration degree in nucleotides out of the conserved hexanucleotide core (5′-CARGGT-3′) and that could be present in the promoter regions of secondary target genes of ZafA.

## Figures and Tables

**Table 1:** *Aspergillus fumigatus* strains used in this study. Genes in brackets were reintroduced into the *A. fumigatus* genome by targeting them at the intergenic region between the AFUA_2G08360 (*pyrG*) and AFUA_2G08350 genes.

Strain	Detailed Genotype	Reference
CEA17	*pyrG1* (auxotrophic PyrG^–^)	[11]
AF14	Wild-type (isogenic to CEA17)	[12]
AF171	Δ*zafA::hisG* (isogenic to CEA17)	[14]
AFZR0	Wild-type [*luc* ← P*zrfC^wt^* → *gfp*] (isogenic to CEA17)	This study
AFZR1	Wild-type [*luc* ← P*zrfC^ZR123^* → *gfp*] (isogenic to CEA17)	This study

**Table 2:** Measurement by RT-qPCR of the expression levels of several genes of each group detected in microarrays, both in a wild-type strain grown in zinc-limiting versus zinc-replete conditions (as in Exp#1) and in a ∆*zafA* strain grown under zinc-limiting conditions with respect to a wild-type strain grown in the same culture conditions (i.e., as in Exp#2). ZafA can be formally considered as an inductor or repressor of the DZR genes, as indicated. The indicated genes (*) should be reallocated into the specified group according to RT-qPCR data, provided that the genes were considered (arbitrarily) to be expressed differentially when their transcription levels changed > 1.5-fold. Expression of genes in red and blue show induction or repression respectively.

Gene ID	Group of Genes	Gene Name	wt − Zn/wt + Zn rER ± SD	∆*zafA* − Zn/wt − Zn rER ± SD	Predicted Effect of ZafA on Gene Expression	Reallocating Subgroup of Genes *
AFUA_1G01550	DZR	*zrfA*	231.3 ± 27.8	−207.7 ± 18.9	Induction	
AFUA_1G02150	Control	*gfdA*	−1.25 ± 0.15	1.17 ± 0.11		
AFUA_1G03150	DZR	*erg24A*	−49.9 ± 7.0	2.0 ± 0.2	Repression	
AFUA_1G04620	IZR	-	−4.9 ± 0.4	−1.9 ± 0.2		
AFUA_1G07480	NZR	*hem13*	−6.0 ± 0.8	−1.6 ± 0.1		IZR
AFUA_1G10060	NZR	-	3.1 ± 0.2	−1.2 ± 0.1		
AFUA_1G10080	DZR	*zafA*	31.5 ± 3.0	−21582 ± 4316	Induction	
AFUA_1G10130	NZR	*sahA*	1.9 ± 0.1	1.1 ± 0.1		
AFUA_1G12830	IZR	*niaD*	−1.6 ± 0.1	−5.2 ± 0.7		
AFUA_1G13510	NZR	*facB*	4.6 ± 0.6	1.0 ± 0.1		
AFUA_1G14550	DZR	*sodC*/*sod3*	1698.7 ± 198.2	−1176.5 ± 142.4	Induction	
AFUA_1G15590	NZR	*cybS*/*sdh4*	2.9 ± 0.4	1.1 ± 0.1		
AFUA_1G17190	NZR	*sidI*	−92.5 ± 8.7	1.1 ± 0.1		
AFUA_2G00320	NZR	*erg3B*	−271.7 ± 31.7	−1.6 ± 0.2		IZR
AFUA_2G01260	DZR	*srbA*	−15.8 ± 2.1	2.1 ± 0.2	Repression	
AFUA_2G03010	NZR	-	−1.7 ± 0.2	−1.0 ± 0.1		
AFUA_2G03700	NZR	*hmg1*	−2.6 ± 0.2	−1.1 ± 0.1		
AFUA_2G03860	DZR	*zrfB*	33.0 ± 2.7	−79.3 ± 7.7	Induction	
AFUA_2G07680	IZR	*sidA*	−27.3 ± 3.3	−6.9 ± 0.6		
AFUA_2G08740	DZR	*zrfF*	24.8 ± 1.8	−16.0 ± 2.0	Induction	
AFUA_2G11120	DZR	*gtmA*	5.7 ± 0.7	−20.3 ± 1.8	Induction	
AFUA_2G14570	NZR	*zrcC*	−2.1 ± 0.2	−1.1 ± 0.1		
AFUA_2G15010	NZR	-	−1.5 ± 0.2	−1.2 ± 0.1		
AFUA_2G15290	DZR	-	−26.0 ± 3.6	3.2 ± 0.3	Repression	
AFUA_2G17550	IZR	*ayg1*	−2.7 ± 0.4	−103.5 ± 10.8		
AFUA_3G02270	DZR	*cat1*	18.9 ± 1.8	−2.0± 0.2	Induction	
AFUA_3G03640	IZR	*mirB*	−3225.8 ± 304.5	−10.0 ± 1.2		
AFUA_3G14440	NZR	-	−3.5 ± 0.5	−1.3 ± 0.1		
AFUA_4G03410	NZR	*fhpA*	−3.1 ± 0.3	−1.4 ± 0.2		
AFUA_4G03460	IZR	*srbB*	−50.4 ± 5.9	−1.1 ± 0.1		NZR
AFUA_4G03930	DZR	*cysX*	12786.9 ± 1492.2	−7751.9 ± 938.0	Induction	
AFUA_4G06530	NZR	*metR*	−1.6 ± 0.2	1.2 ± 0.1		
AFUA_4G09560	DZR	*zrfC*	1270.1 ± 108.0	−5377.1 ± 424.8	Induction	
AFUA_4G10730	Control	*rvb1*	1.1 ± 0.2	−1.2 ± 0.1		
AFUA_4G12840	IZR	-	−1.6 ± 0.2	−1.7 ± 0.1		
AFUA_5G01030	DZR	*gpdB*	−1.2 ± 0.1	77.7 ± 7.5		SSA
AFUA_5G01970	NZR	*gpdA*	1.8 ± 0.2	1.0 ± 0.1		
AFUA_5G02180	NZR	*cysB*	−2.6 ± 0.2	1.1 ± 0.1		
AFUA_5G03800	NZR	*ftrA*	−31.0 ± 2.2	1.4 ± 0.2		
AFUA_5G03920	NZR	*hapX*	−12.2 ± 1.5	−1.2 ± 0.1		
AFUA_5G04130	Control	*phoA*	1.1 ± 0.1	−1.3 ± 0.2		
AFUA_5G06240	DZR	*alcC*	−26.8 ± 1.9	3.8 ± 0.5	Repression	
AFUA_5G06270	NZR	*hemA*	1.8 ± 0.2	−1.0 ± 0.1		
AFUA_5G08090	DZR	*pyroA*	2.7 ± 0.3	−1.8 ± 0.2	Induction	
AFUA_5G09240	DZR	*sodA*/*sod1*	−4.7 ± 0.7	7.3 ± 0.8	Repression	
AFUA_5G09360	Control	*calA*	1.1 ± 0.1	1.1 ± 0.1		
AFUA_5G09680	NZR	*carC*/*sdh3*	3.9 ± 0.4	−1.0 ± 0.1		
AFUA_5G10560	Control	*cox5A*	−1.2 ± 0.2	1.0 ± 0.1		
AFUA_6G00690	DZR	-	−2.8 ± 0.3	5.2 ± 0.6	Repression	
AFUA_6G04430	NZR	-	2.0 ± 0.2	1.3 ± 0.2		
AFUA_6G04740	Control	*actA*	−1.0 ± 0.1	1.2 ± 0.1		
AFUA_6G05160	DZR	*azf1*	1.9 ± 0.2	−23.9 ± 2.9	Induction	
AFUA_6G07720	IZR	*acuF*	−2.2 ± 0.2	−1.7 ± 0.2		
AFUA_6G09630	DZR	*gliZ*	45.9 ± 6.0	−67.2 ± 5.3	Induction	
AFUA_6G09710	DZR	*gliA*	3548.5 ± 87.4	−3039.5 ± 294.8	Induction	
AFUA_6G09740	DZR	*gliT*	53.6 ± 3.9	−477.3 ± 59.7	Induction	
AFUA_7G00250	IZR	*tubB2*	−7.0 ± 0.5	−10.0 ± 1.2		
AFUA_7G02560	Control	*dld1*	−1.1 ± 0.1	1.0 ± 0.1		
AFUA_7G04730	IZR	*enb1*	−48.3 ± 5.8	−2.4 ± 0.2		
AFUA_7G05070	NZR	*frdA*	−56.5 ± 7.4	1.9 ± 0.1	Repression	DZR
AFUA_7G06570	DZR	*zrcA*	−5.9 ± 0.7	16.6 ± 1.5	Repression	
AFUA_7G06790	DZR	*yct1*	6039.9 ± 362.4	−5555.6 ± 577.8	Induction	
AFUA_8G02450	DZR	*gzbA*	84.8 ± 8.0	−275.5 ± 32.2	Induction	
AFUA_8G02620	DZR	*mchC*	1155.6 ± 134.9	−524.3 ± 63.4	Induction	

SSA: strain-specific adapted (genes); DZR: directly ZafA regulated; IZR: indirectly ZafA regulated; NZR: non-ZafA regulated.

**Table 3:** Genes regulated or that were predicted to be regulated by ZafA. The expression of genes in red and blue was shown to be induced and repressed respectively by ZafA, as confirmed by RT-qPCR and/or northern-blot. The expression of genes in **black** and green was predicted to be induced and repressed respectively by ZafA. Start and end positions of the ZR motifs were numbered taking as reference that the base preceding the A nucleotide of the ATG translation start codon is at position −1. Motifs labeled with an asterisk (*) had two substitutions with respect to the ZR consensus motif out of the hexanucleotide core sequence 5′-CARGGC-3′ (ZR0*).

Gene ID	Gene Name	Strand	Start	End	Sequence ZR Motif	Type of ZR Motif
AFUA_1G01550	*zrfA*	D	−75	−61	GACCTCAAGGTACCC	ZR2
		R	−105	−91	ACTGCCAAGGTCCTT	ZR0
		D	−157	−143	GCTCACAAGGTGTGT	ZR0*
AFUA_1G05900	-	D	−393	−379	TTCGTCAAGGTTGTT	ZR0*
AFUA_1G09810	-	R	−168	−154	ACTCCCAGGGTACTT	ZR0
AFUA_1G10080	*zafA*	D	−361	−347	GCTCCCAAGGTCCTC	ZR0
		D	−448	−434	CCCCTCAGGGTATTA	ZR0*
		D	−624	−610	GCTCGCAGGGTCCTC	ZR5
		D	−657	−643	TCCCCCAGGGTCCTC	ZR0
AFUA_1G12170	*-*	R	−364	−350	ATTACCAAGGTGATA	ZR0*
AFUA_1G12850	*crnA*	R	−500	−486	TGTCCCAGGGTGTAC	ZR0*
AFUA_1G14550	*sodC*/*sod3*	R	−123	−109	AGCATCAAGGTCCTC	ZR2
		D	−160	−146	GATCCCAAGGTCCCC	ZR2
AFUA_1G14560	*msdS*	D	−664	−650	TCTACCAGGGTATAT	ZR8
		D	−928	−914	ATCGCCAGGGTCTTA	ZR9
AFUA_1G14700	*-*	R	−181	−167	GCACACAAGGTACTC	ZR0*
AFUA_2G01260	*srbA*	D	−886	−872	TTGGTCAAGGTCCTT	ZR3
AFUA_2G01610	*-*	D	−841	−827	GTCAGCAGGGTCATC	ZR0*
AFUA_2G02950	*-*	D	−160	−146	GTCTCCAAGGTCTCC	ZR4
AFUA_2G03860	*zrfB*	R	−359	−345	ATCCTCAAGGTCCCT	ZR0
		D	−496	−482	GCCACCAAGGTCCTT	ZR0
		D	−794	−780	TCTCCCAAGGTCCCC	ZR0
		D	−893	−879	ACTCCCAAGGTCCTC	ZR0
AFUA_2G06140	*-*	R	−461	−447	GTTCCCAAGGTCTCC	ZR0
AFUA_2G07810	*-*	D	−756	−742	GTAGGCAAGGTGCCT	ZR0*
AFUA_2G08280	*maeA*	R	−910	−896	TTCACCAGGGTAGAC	ZR0*
AFUA_2G08740	*zrfF*	R	−329	−315	TCTCCCAAGGTCCGC	ZR8
		R	−616	−602	ATAGCCAAGGTAGCT	ZR0*
AFUA_2G15290	*-*	R	−68	−54	GTCAACAGGGTGCTC	ZR5
AFUA_3G02270	*cat1*	D	−1052	−1038	TTGACCAGGGTCTCT	ZR3
AFUA_3G10680	*-*	R	−179	−165	GATCCCAAGGTCCTT	ZR2
		D	−233	−219	TCCCCCAGGGTCCCC	ZR0
AFUA_3G13100	*-*	D	−581	−567	TTCGTCAAGGTTTCA	ZR0*
AFUA_3G13940	*-*	D	−1073	−1059	TTTTCCAAGGTGTTG	ZR0 *
AFUA_4G03920	*-*	R	−307	−293	CTCACCAAGGTCCCC	ZR1
		D	−438	−424	ACCTCCAAGGTCCTG	ZR0*
		R	−576	−562	TCTATCAAGGTAATT	ZR7
		R	−605	−591	GCCTCCAAGGTAGTC	ZR0*
AFUA_4G03930	*cysX*	D	−272	−258	GCCTCCAAGGTAGTC	ZR0*
		D	−301	−287	TCTATCAAGGTAATT	ZR1
		R	−439	−425	ACCTCCAAGGTCCTG	ZR0*
		D	−570	−556	CTCACCAAGGTCCCC	ZR7
AFUA_4G09560	*zrfC*	R	−196	−182	ACTCCCAAGGTATCT	ZR0
		R	−313	−299	GCTCTCAAGGTCCTC	ZR0
		R	−637	−623	GCTACCAAGGTGTCC	ZR0
		D	−749	−735	GCCATCAGGGTAGAC	ZR0*
AFUA_4G09580	*aspf2*	R	−149	−135	GCCATCAGGGTAGAC	ZR0*
		D	−261	−247	GCTACCAAGGTGTCC	ZR0
		D	−585	−571	GCTCTCAAGGTCCTC	ZR0
		D	−702	−688	ACTCCCAAGGTATCT	ZR0
AFUA_4G10460	*hcsA*	R	−538	−524	ATCGGCAAGGTACAT	ZR0*
AFUA_5G02010	*-*	D	−122	−108	TTCTCCAGGGTCTTA	ZR0*
AFUA_5G03060	*-*	R	−807	−793	GCGCGCAAGGTACTT	ZR0*
AFUA_5G05710	*-*	R	−100	−86	TTCACCAAGGTTTTG	ZR0*
AFUA_5G06240	*alcC*	D	−595	−581	TCCCCCAGGGTACAT	ZR8
AFUA_5G09240	*sodA*/*sod1*	R	−198	−184	ATTCACAGGGTATTA	ZR0*
AFUA_5G12780	*-*	D	−99	−85	ACTCCCAAGGTACTC	ZR0
AFUA_5G13940	*-*	R	−231	−217	TCTGTCAGGGTCTGT	ZR8
AFUA_6G00690	*-*	D	−77	−63	GTCCACAAGGTCTTC	ZR5
AFUA_6G08580	*fkbp4*	D	−134	−120	GCCCTCAAGGTTCCT	ZR6
AFUA_6G09310	*-*	D	−1068	−1054	AGTGACAAGGTATTC	ZR0*
AFUA_6G09630	*gliZ*	D	−790	−776	TCTAACAAGGTCCTC	ZR5
		D	−836	−822	GCCCCCAAGGTGCCT	ZR0
AFUA_6G09710	*gliA*	R	−469	−455	GTTTGCAAGGTACTC	ZR0*
		R	−522	−508	TCCCCCAAGGTCACA	ZR0*
AFUA_6G10260	*akr1*	D	−180	−166	GTCGTCAAGGTTCCC	ZR6
AFUA_7G02360	*-*	R	−323	−309	TCGATCAAGGTGCTT	ZR3
AFUA_7G03970	*-*	D	−396	−382	TGTACCAAGGTATGT	ZR0*
AFUA_7G06570	*zrcA*	D	−915	−901	GTCCCCAAGGTACTC	ZR0
AFUA_7G06790	*yct1*	D	−224	−210	GTTCACAAGGTTCTT	ZR0*
		D	−305	−291	TCTACCAAGGTCCTT	ZR0
AFUA_7G06810	*sarA*	R	−187	−173	TCCAACAAGGTACCT	ZR5
		R	−276	−262	CCTCCCAGGGTTCTC	ZR0*
AFUA_8G01930	-	R	−603	−589	GCCATCAAGGTCGAT	ZR0*
AFUA_8G02450	*gzbA*	R	−165	−151	GTCTTCAAGGTTCTC	ZR0*
		D	−401	−387	GTCCCCAAGGTTCTC	ZR6
AFUA_8G02460	*-*	R	−617	−603	GTCCCCAAGGTTCTC	ZR6
		D	−853	−839	GTCTTCAAGGTTCTC	ZR0*
AFUA_8G02620	*mchC*	R	−65	−51	ACCCCCAAGGTTCGC	ZR0*
		R	−172	−158	ACTCCCAAGGTACCT	ZR0
		R	−192	−178	GGGCTCAAGGTCCTC	ZR0*

## Supplementary Materials

The following are available online at http://www.mdpi.com/2073-4425/9/7/318/s1, Table S1. Oligonucleotides used for RT-qPCR. Table S2. Oligonucleotides used in this study for construction of plasmids and validation of fungal strains. Table S3. Genes differentially expressed in a wild-type strain grown in zinc-limiting (SDN–Zn) versus zinc-replete conditions (SDN + 100 µM Zn) (Exp#1; FC > 1.5; *P* < 0.05). Table S4. Genes differentially expressed in a ∆*zafA* strain grown under zinc-limiting conditions with respect to a wild-type strain grown in the same culture conditions (Exp#2; FC > 1.5; *P* < 0.05). Table S5. Genes up-regulated in Exp#1 but down-regulated in Exp#2 and vice versa (i.e., DZR genes). Table S6. Functional categorization of genes differentially expressed in a wild-type strain grown in zinc-limiting (SDN–Zn) versus zinc-replete conditions (Exp#1). Table S7. Control genes used for MEME analysis. Table S8. All possible ZR motifs harboring one substitution out of the conserved hexanucleotide core (5′-CARGGT-3′) with respect to the ZR consensus motif (ZR0). Figure S1. Construction of strains used to show the function of ZafA in regulating gene expression through binding to the ZR motifs in vivo. Figure S2. Analysis of ZafA binding to DNA fragments from the promoter regions of genes *zafA*, *zrfA*, *zrfB* and *zrfC* by DNase I footprinting assays.

## Fundings

This work was supported by the “Ministerio de Economía y Competitividad” (Spain) and “Junta de Castilla y León” (Spain) through grants SAF2013-48382-R and SA020G18 respectively to J.A.C.
