# Supplementary material for: The Transcription Factor ZafA Regulates the Homeostatic and Adaptive Response to Zinc Starvation in Aspergillus fumigatus
Source: Genes (Basel). 2018 Jun 26;9(7):318. doi: 10.3390/genes9070318 (PMC6070888; doi:10.3390/genes9070318)
Supplement: Supplementary file 1 [file genes-09-00318-s001.zip › Supplementary files/Fig S1.pdf]

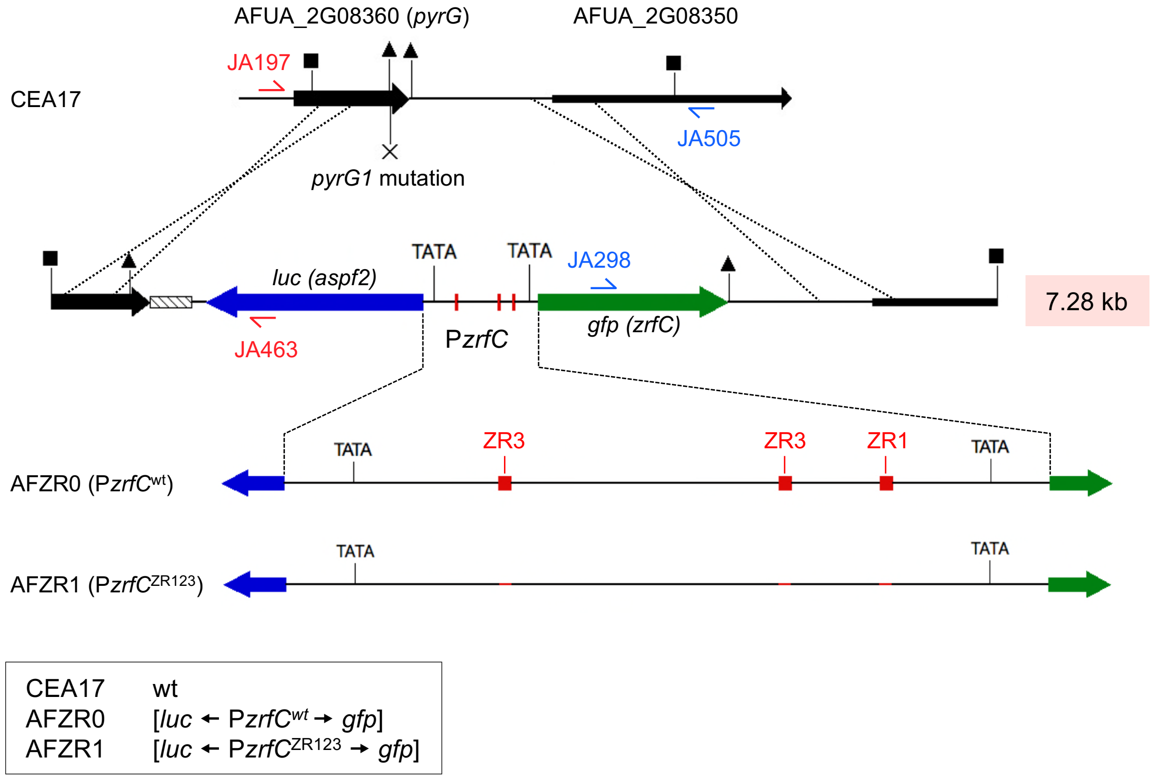

Figure S1. Construction of strains used to show the function of ZafA in regulating gene expression through binding to the ZR motifs *in vivo*. Schematic representation of the construction of the derivative uridine-uracil-prototrophic PyrG^{+} *A. fumigatus* strains AFZR0 and AFZR1 that harbored the coding sequence of the firefly luciferase (*luc*, blue arrow) and green fluorescent protein (*gfp*, green arrow) under the control of the divergent *aspf2-zrfC* promoter (abbreviated as P*zrfC*). The three ZR motifs present in P*zrfC* (red boxes) had been inactivated by site directed mutagenesis in the mutant version of this promoter (P*zrfC*^{ZR123}). EcoRI-EcoRI DNA fragments of 7286 bp excised from plasmids pPYRGQ191 and pPYRGQ193, which carried respectively the [*luc* ← P*zrfC*^{wt} → *gfp*] and [*luc* ← P*zrfC*^{ZR123} → *gfp*] constructs, were used to transform the CEA17 uridine-uracil-auxotrophic *pyrG1* strain to generate respectively the AFZR0 and AFZR1 strains. Both strains harboured the DNA fragment of interest inserted at the *pyrG* locus between their AFUA_2G08360 (*pyrG*) and AFUA_2G08350 loci, as verified by PCR analyses using the pairs of oligonucleotides JA197/JA505, JA197/JA463 and JA298/JA505.
