# Supplementary material for: The Transcription Factor ZafA Regulates the Homeostatic and Adaptive Response to Zinc Starvation in Aspergillus fumigatus
Source: Genes (Basel). 2018 Jun 26;9(7):318. doi: 10.3390/genes9070318 (PMC6070888; doi:10.3390/genes9070318)
Supplement: Supplementary file 1 [file genes-09-00318-s001.zip › Supplementary files/Fig S2.pdf]

Figure S2. Analysis of ZafA binding to DNA fragments from the promoter regions of genes *zafA*, *zrfA*, *zrfB* and *zrfC* by DNase I footprinting assays. The purple chromatograms were obtained upon incubating the DNA fragment in the absence of ZafA^{ZF3-6}, such that the DNA remained naked and, hence, unprotected from DNase I digestion. The yellow chromatogram were obtained upon incubating the DNA in the presence of ZafA^{ZF3-6}. In this case, ZafA bound to DNA prevented its digestion with DNase I resulting in a stretch of relatively low yellow peaks (compared to the high of the control purple peaks) that was typically followed by a hypersensitive region to DNase I digestion, as reflected by a short stretch of high yellow peaks typically observed in most cases at the right of the chromatograms. The protected regions in the chromatograms are delimited by a bracket. Every ZR protected sequence of each gene has been named and numbered properly either in black letters (for sequences in the direct strand) or in green letters followed by a comma (for sequences in the reverse-complementary or antiparallel strand). Below each chromatogram is shown a schematic representation of the whole intergenic region of the corresponding gene with the beginning of its ORF located at the right side (green arrow). The sequence of the DNA fragments used in the assay corresponding to the promoter sequence is indicated in the scheme with a black rectangle and shown at the nucleotide level at the bottom of each set of chromatograms. In these sequences their putative ZR motifs detected in MEME have been highlighted in red or gray. The protected regions are indicated in the schemes with a green rectangle. The yellow stars indicate the end 6-FAM labeled strand detected by the capillary-based automated DNA sequencer.

Fig. S2

zafA promoter

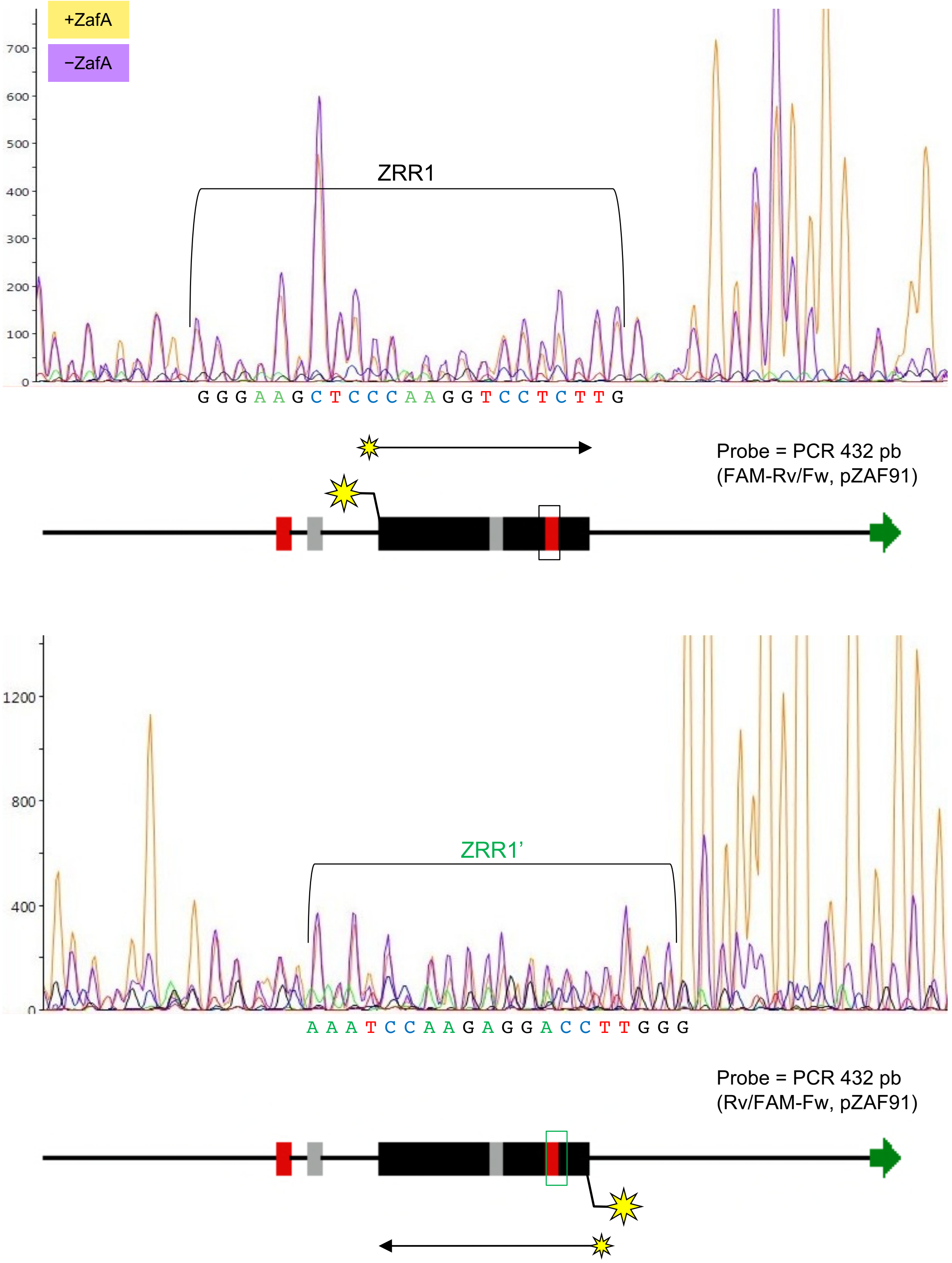

Sequence of the P*zafA* fragment (229 bp) within the probe:

```
CACGCGCCCATATGCAAGAATCTTATTGAGGTACGTATTATTCTAGGCTCTAATGCGAGTCCTGCACTA
ATCCTCATGTCTAGGTAGTCCCCTGTCCCCTCAGGGTATTACCAGGGCCCCTTTCCCTCAAGTTGCTCG
CACAGATCTGGATGTTACTTTCATCGAATATATACACGTGGGAAGCTCCCAAGGTCCTCTTGGATTTGC
ACACTGCAGGCGGTCAATCCTA...N314...ATG
```

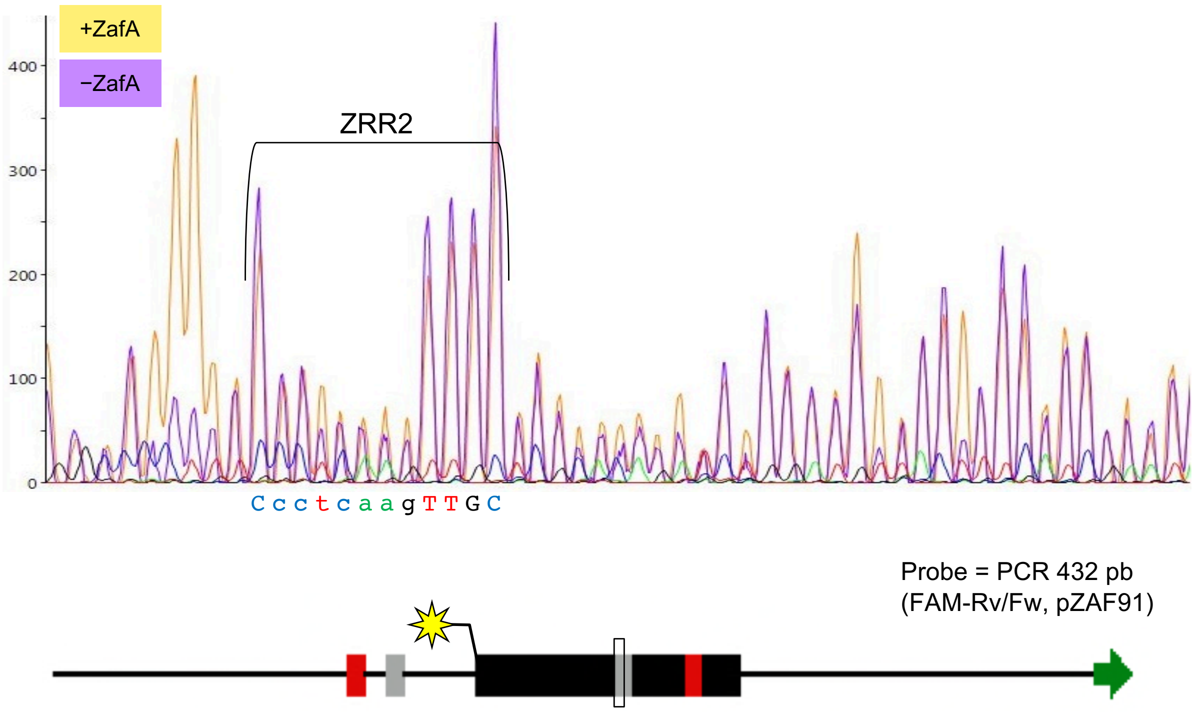

Sequence of the P*zafA* fragment (229 bp) within the probe:

CACGCGCCCATATGCAAGAATCTTATTGAGGTACGTATTATTCTAGGCTCTAATGCGAGTCCTGCACTA
ATCCTCATGTCTAGGTAGTCCCCTGTCCCCTCAGGGTATTACCAGGGCCCCTTTCCCTCAAGTTGCTCG
CACAGATCTGGATGTTACTTTCATCGAATATATACACGTGGGAAGCTCCCAAGGTCCTCTTGGATTTGC
ACACTGCAGGCGGTCAATCCTA...N_{314}...ATG

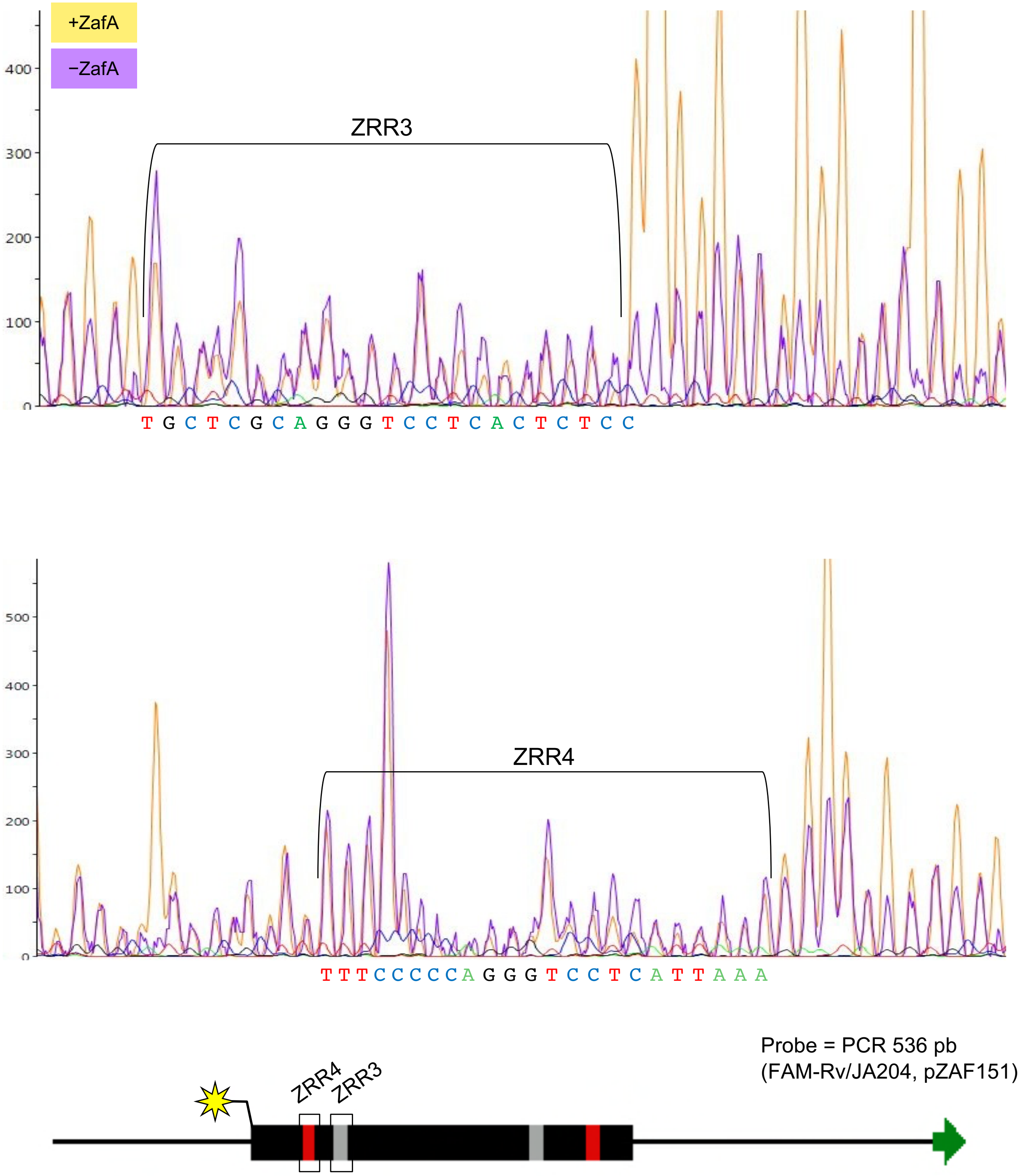

Sequence of the P*zafA* fragment (396 bp) within the probe:

CCTGACAATTCAGGCGATACGATTCGCCGGCGGGGAGTGGGCATGACGCTTTTTCCCCCAGGGTCCTCA
TTAAAGAATAGGTGCTTGCTCGCAGGGTCCTCACTCTCCTTCTTGCTTGTGCGTGATAGAATCCGGTCG
CTGTGTCTGGATGGGGCCAGGTAGCACCCCACGCGCCCATATGCAAGAATCTTATTGAGGTACGTATTA
TTCTAGGCTCTAATGCGAGTCCTGCACTAATCCTCATGTCTAGGTAGTCCCCTGTCCCCTCAGGGTATT
ACCAGGGCCCCTTTCCCTCAAGTTGCTCGCACAGATCTGGATGTTACTTTCATCGAATATATACACGTG
GGAAGCTCCCAAGGTCCTCTTGGATTTGCACACTGCAGGCGGTCAATCCTA...N_{314}...ATG

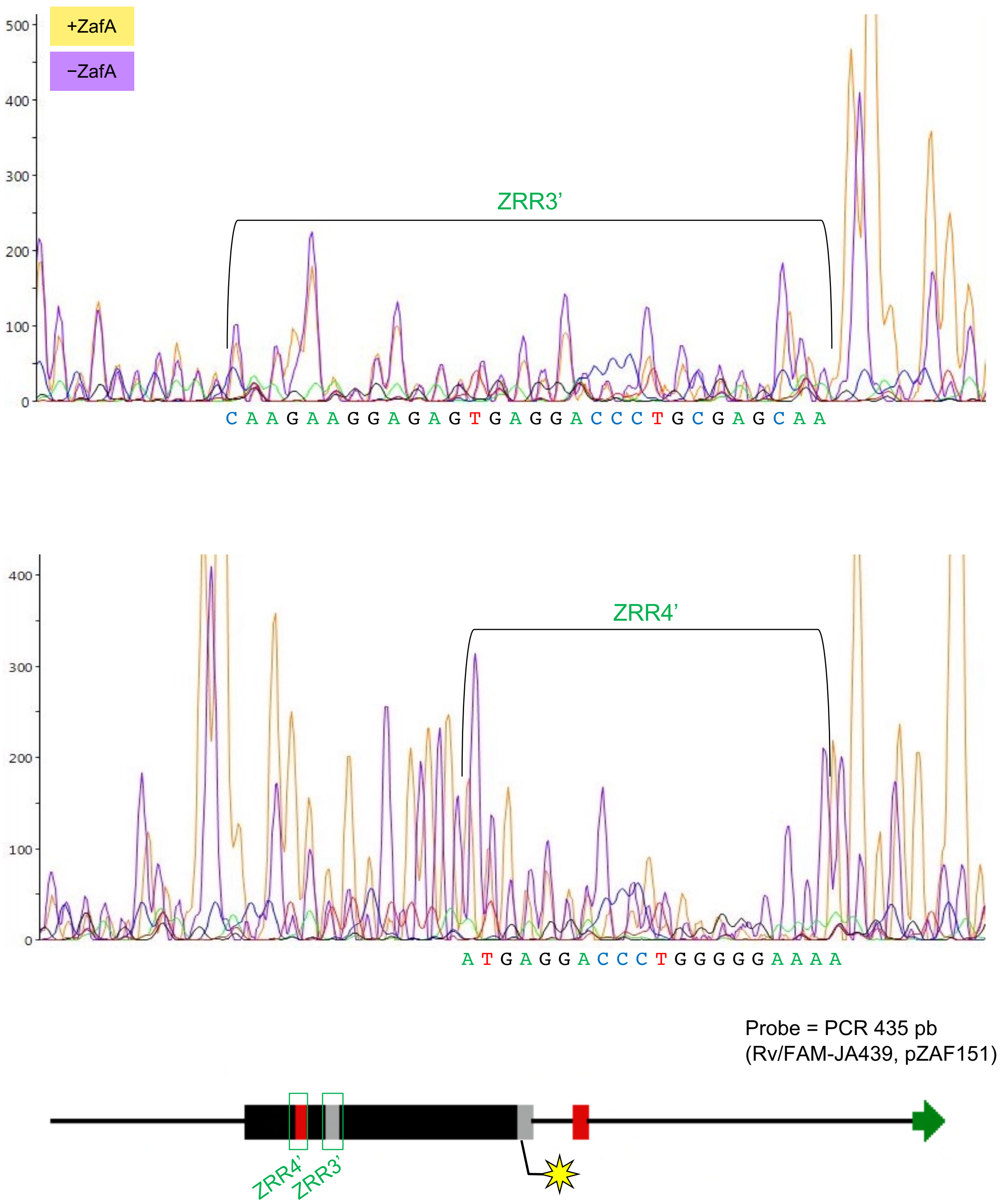

Sequence of the P*zafA* fragment (295 bp) within the probe:

CCTGACAATTCAGGCGATACGATTCGCCGGCGGGAGTGGGCATGACGCTTTTTCCCCCAGGGTCCTCA
TTAAAGAATAGGTGCTTGCTCGCAGGGTCCTCACTCTCCTTCTTGCTTGTGCGTGATAGAATCCGGTCG
CTGTGTCTGGATGGGGCCAGGTAGCACCCCACGCGCCCATATGCAAGAATCTTATTGAGGTACGTATTA
TTCTAGGCTCTAATGCGAGTCCTGCACTAATCCTCATGTCTAGGTAGTCCCCTGTCCCCTCAGGGTATT
ACCAGGGCCCCTTTCCCTC...N_{415}...ATG

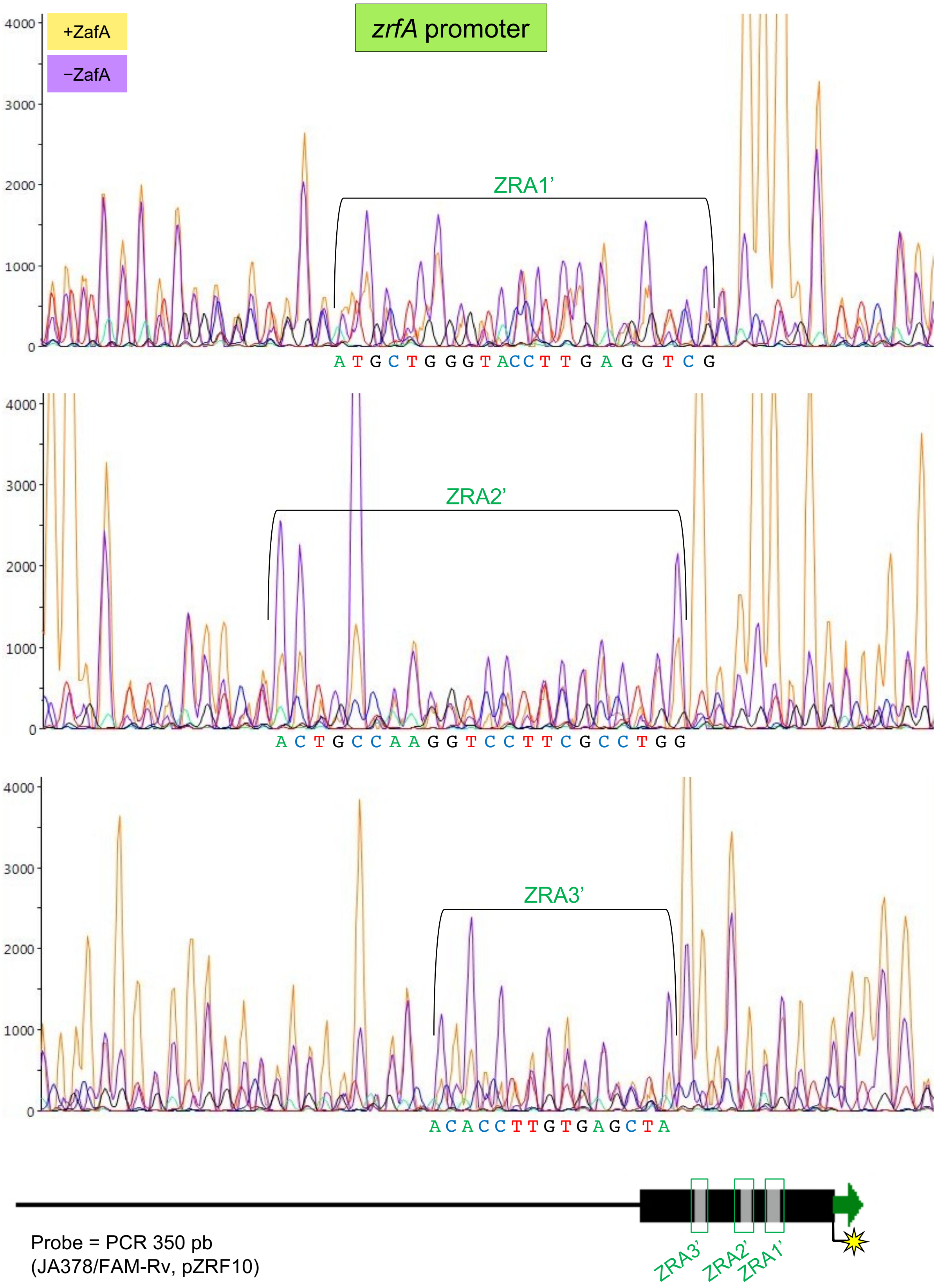

Sequence of the P*zrfA* fragment (217 bp) within the probe:

ACAGGCTAACGCTTGTGATGACACTTTTCTTTCTCGTCTTGCCCACAGGAGCTAGAGGTAGCTCACAAG
GTGTGTCTACTCTCGACCATCACCGCGTCCCACCGACCAGGCGAAGGACCTTGGCAGTAGACTGAATCA
GTGCGACCTCAAGGTACCCAGCATGCCAGCGCCATATAAAGGAAACAGTGTCTAGCACTTCGATGCGGA
TGATGCAACCATG

zrfB promoter

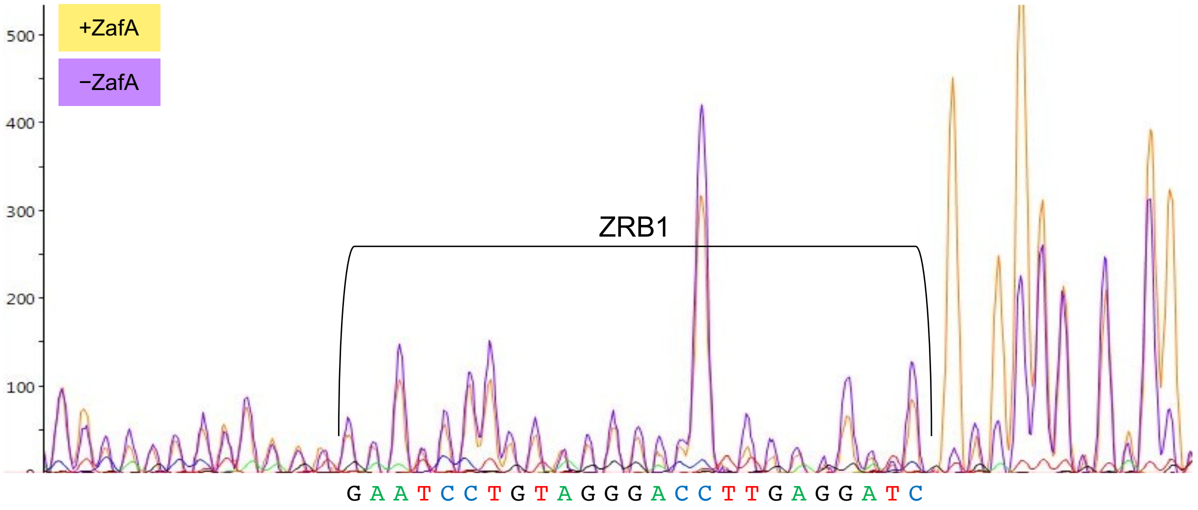

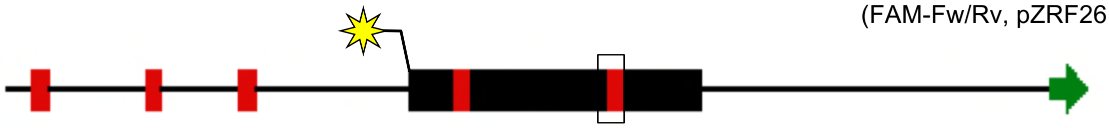

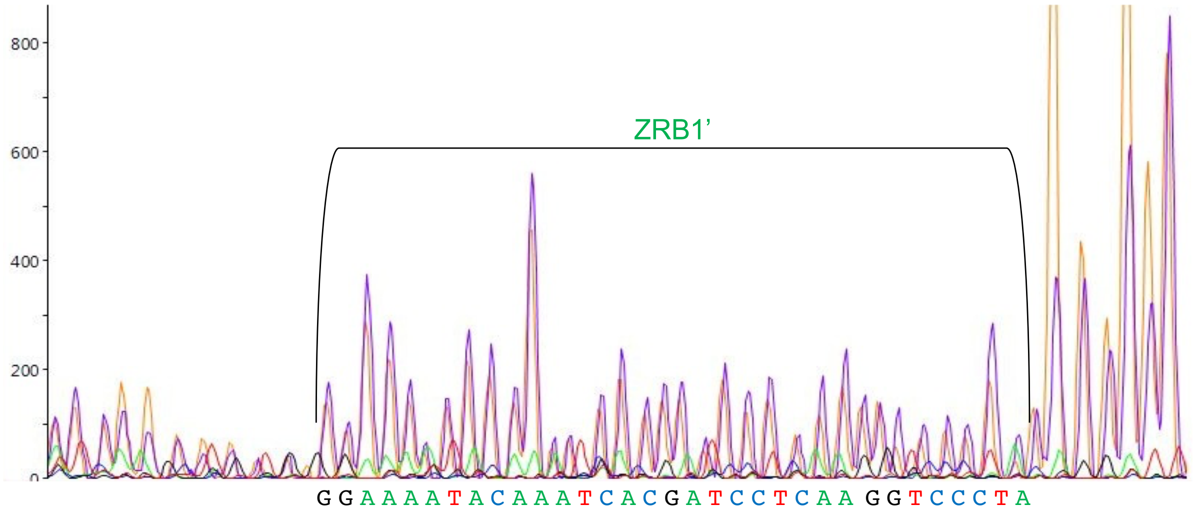

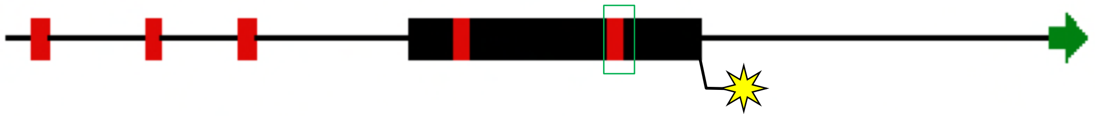

Sequence of the P*zrfB* fragment (256 bp) within the probe:

TCGGACATAACGAACGACTCATTGGGGATCGCAGAATACCTGCTTGCCTGCTGGGTTTCTTGCCTTGCC
ACCAAGGTCCTCAATTGCGGATTCAAAATCCCTGCTACTGCGTTAACTGCGAGCGATCACACGACAGTC
AAGCCTCTGACCTCGTTCTTATCACAGGCTTAGAAGTCTTAGTACTCAGCCTAAGTGAATCCTGTAGGG
ACCTTGAGGATCGTGATTTGTATTTTCCCACAGCTTGATCCAAGGGAGT...N_{306}...ATG

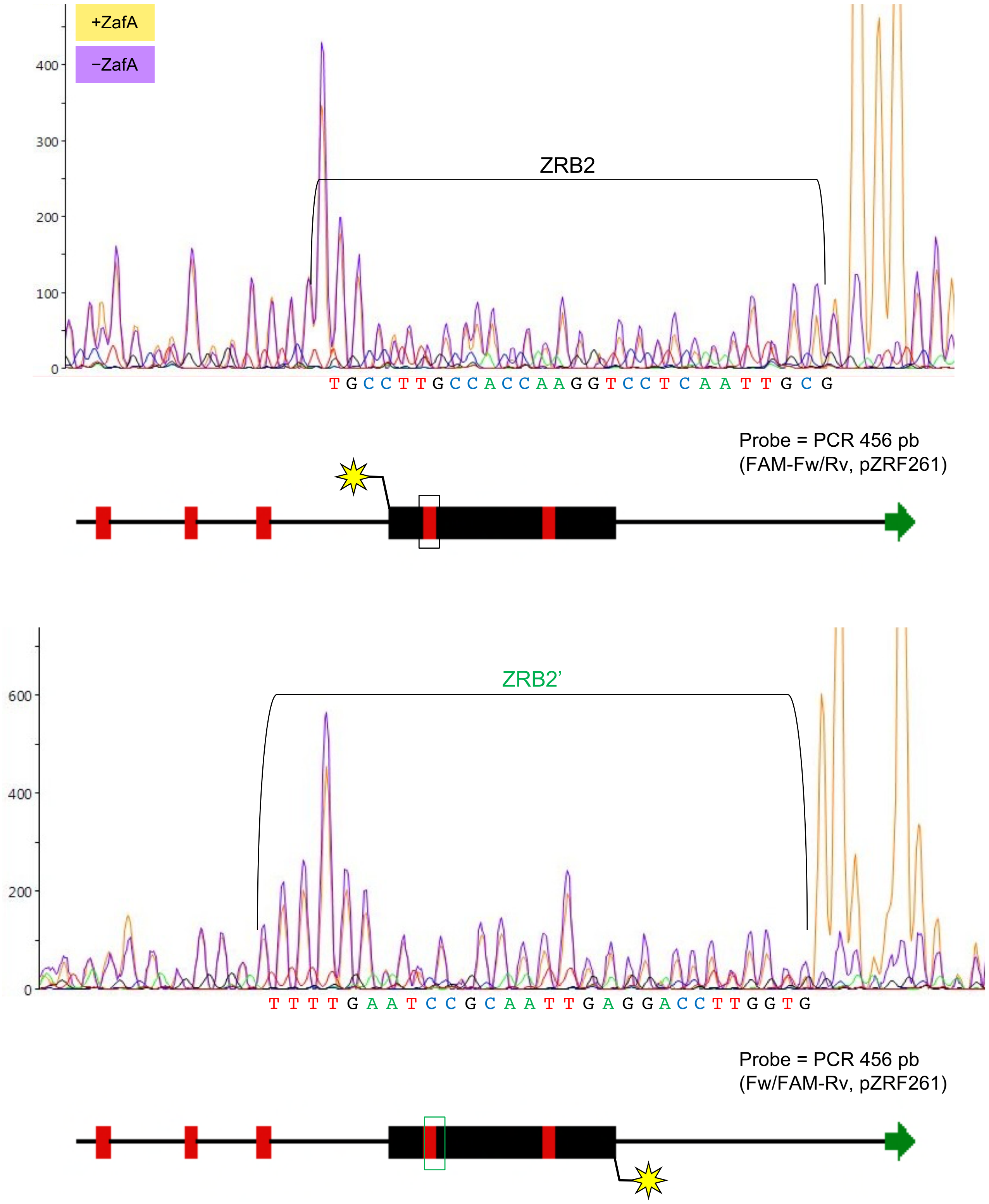

Sequence of the P*zrfB* fragment (256 bp) within the probe:

TCGGACATAACGAACGACTCATTGGGGATCGCAGAATACCTGCTTGCCTGCTGGGTTTCTTGCCTTGCC
ACCAAGGTCCTCAATTGCGGATTCAAAATCCCTGCTACTGCGTTAACTGCGAGCGATCACACGACAGTC
AAGCCTCTGACCTCGTTCTTATCACAGGCTTAGAAGTCTTAGTACTCAGCCTAAGTGAATCCTGTAGGG
ACCTTGAGGATCGTGATTTGTATTTTCCCACAGCTTGATCCAAGGGAGT...N_{306}...ATG

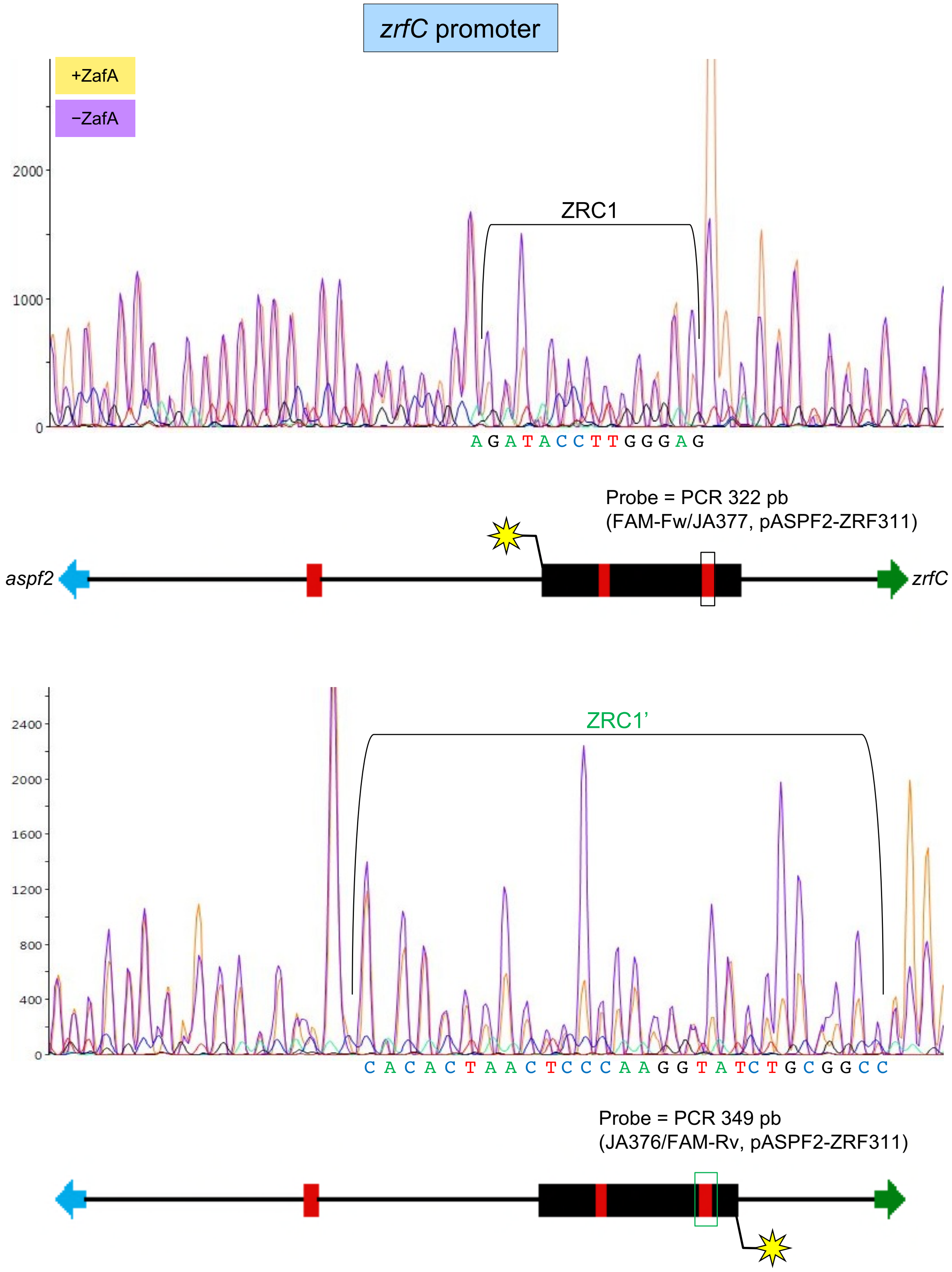

Sequence of the P*zrfC* fragment (215 bp) within the probe:

CAATGCATGGAGGCAGCCCGCGCCAAGATCCTGGCGGAGGAGCCAAGAGTCTTGGATCGAGGACCTTGA
GAGCAACAAGCACCACTCCCGATCATTTTGCTCCGGGAATATAGCCGCTTTCTCGCCGAATGGACGGAG
TTTTGCTGCTTGGCCGTCAGATTGTGCTCTTGGCCGCAGATACCTTGGGAGTTAGTGTGGTGTGTTCAC
GGCGAAAG...N$_{156}$...ATG

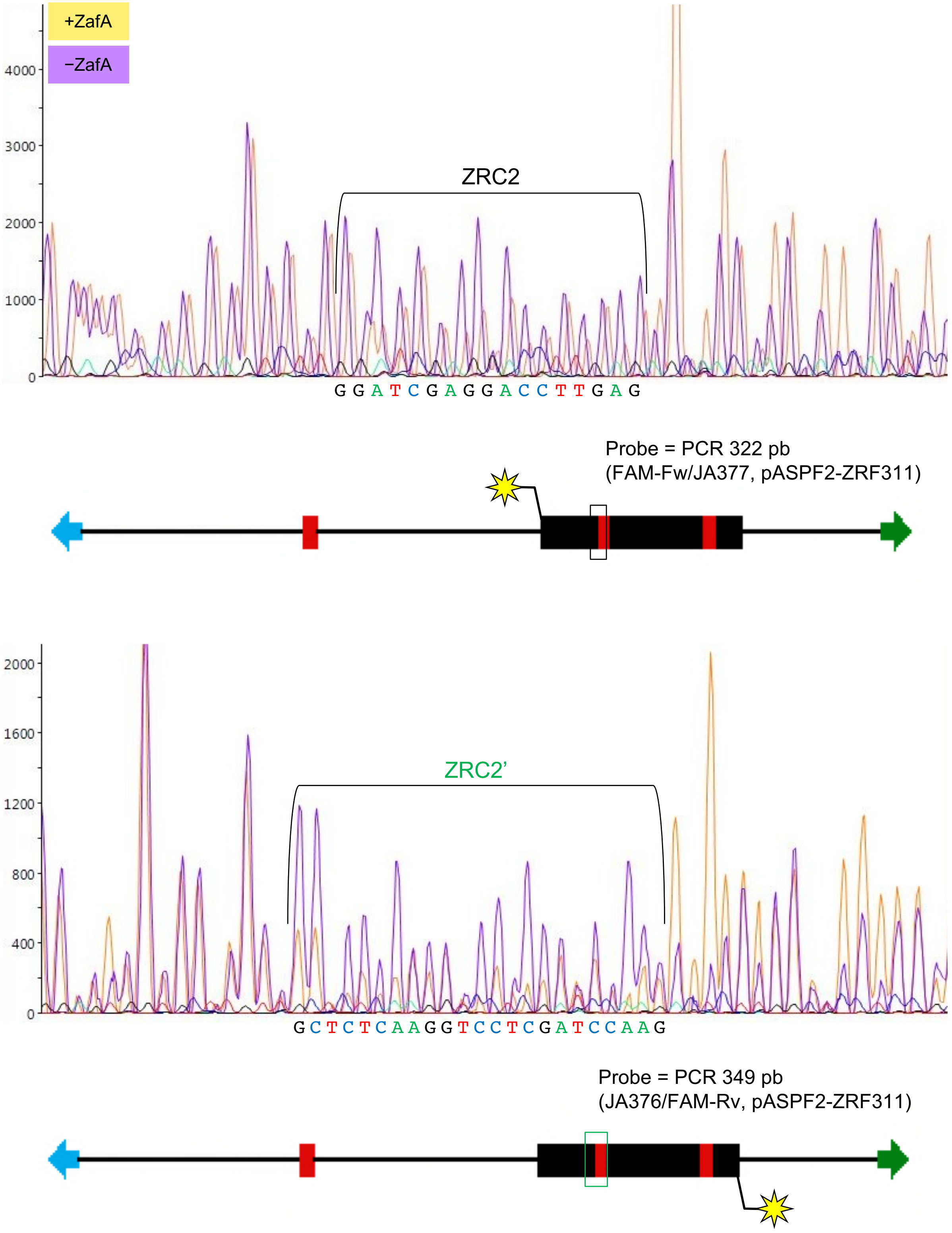

Sequence of the P*zrfC* fragment (215 bp) within the probe:

CAATGCATGGAGGCAGCCCGCGCCAAGATCCTGGCGGAGGAGCCAAGAGTCTTGGATCGAGGACCTTGA
GAGCAACAAGCACCACTCCCGATCATTTTGCTCCGGGAATATAGCCGCTTTCTCGCCGAATGGACGGAG
TTTTGCTGCTTGGCCGTCAGATTGTGCTCTTGGCCGCAGATACCTTGGGAGTTAGTGTGGTGTGTTCAC
GGCGAAAG...N_{156}...ATG

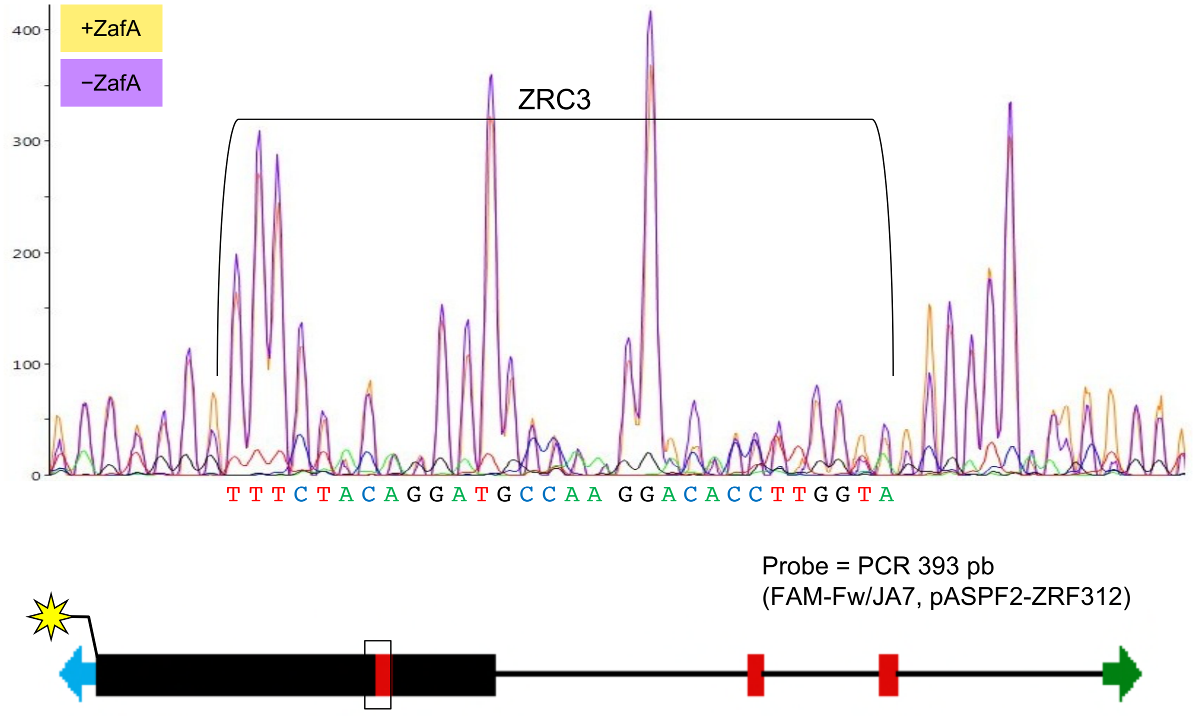

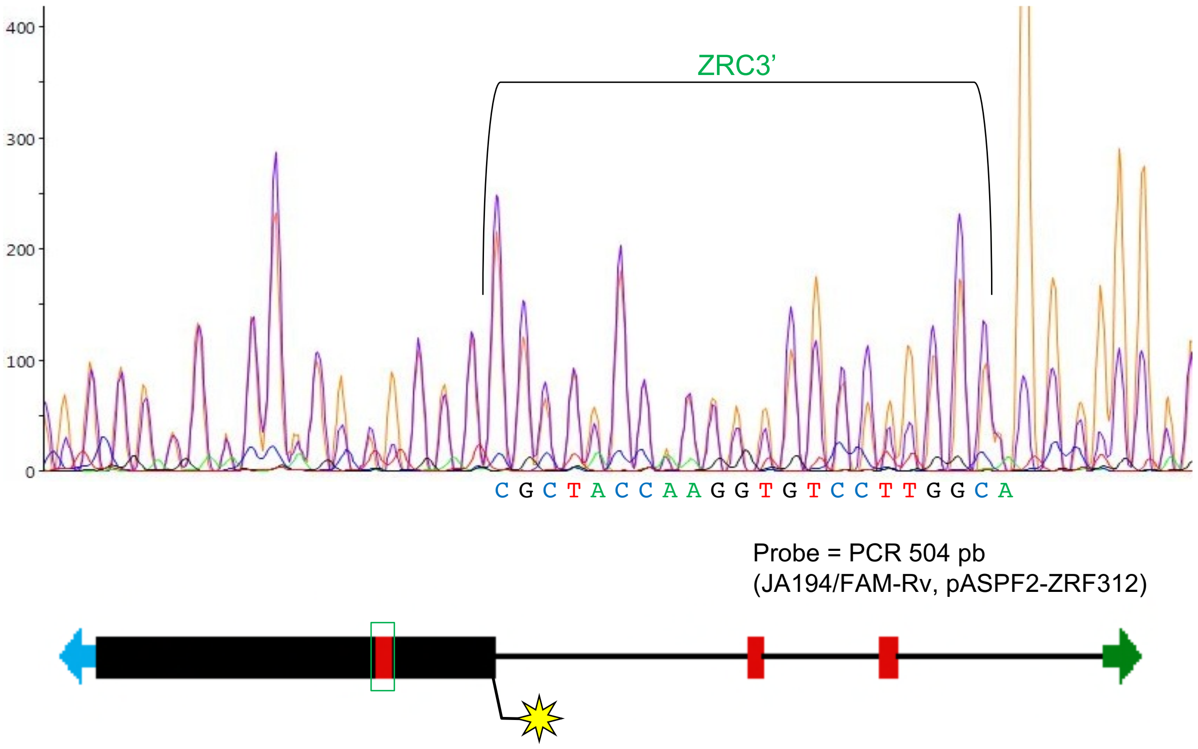

Sequence of the P*zrfC* fragment (349 bp) within the probe:

CATTTGTGAGTACATTTTCTTTTTTCAAGGTTGGATGGTGCGTGACTCTTGCATTGTAGCCAGAGGTAG
AACATCCTTATATACCTCGAGGATGGCCTCCTTTCGCGGTGCCAGAGAGGTACTCATTGTTGGATTTGG
GCCATCAGGGTAGACACGCCACCGGCCCACCCCAACAGTAGGTCACGGCCGCAAGCCATCAAGATGCAT
GGTTCGTTTGTTCGTATTCTAGTGGGTTTCTACAGGATGCCAAGGACACCTTGGTAGCGATCAAGCTGG
TTCTCGAGTCGGATGATTCCGAAAGCTATGATCTCCGAGCTTCTGTCAGCCCTATATGATCCTGTCCGT
ACGTGAT...N_{535}...ATG
