# Supplementary material for: The Transcription Factor ZafA Regulates the Homeostatic and Adaptive Response to Zinc Starvation in Aspergillus fumigatus
Source: Genes (Basel). 2018 Jun 26;9(7):318. doi: 10.3390/genes9070318 (PMC6070888; doi:10.3390/genes9070318)
Supplement: Supplementary file 1 [file genes-09-00318-s001.zip › Supplementary files/Table S1.pdf]

Table S1. Oligonucleotides used for RT-qPCR

Gene ID	Oligonucleotide	5'-sequence-3'
18S rRNA	18SRNA-D	TGTTAAACCCTGTCGTGCTG
18S rRNA	18SRNA-R	GTACAAAGGGCAGGGACGTA
AFUA_1G01550	ZRFA-D1	TGATATACAGTGGGTTGGTCGAGTTGC
AFUA_1G01550	ZRFA-R1	ATCCCAGCTCCCAGGAGTGTAC
AFUA_1G02150	GFDA-D	TCTTTGGTGCCACTATCGACACGAG
AFUA_1G02150	GFDA-R	GATGGGCTGCTTCCTCTCAATGC
AFUA_1G03150	ERG24A-D	GTACATCCAGACGTCTAGTGGCTCG
AFUA_1G03150	ERG24A-R	TGGGCAAGGAATATGACCATGACATCAG
AFUA_1G04620	PODH-D	TCCGCTCAGTTCGAGAAGCTCAAG
AFUA_1G04620	PODH-R	ACACGGTCCTTGTTGGCGTACAC
AFUA_1G07480	HEM13-D	AAGCGCAAGGACATGCCCTTCAC
AFUA_1G07480	HEM13-R	GCAAGCTCATCAAGATACTCTCGACACG
AFUA_1G10060	AHPA-D	CAGCGACCACATACAAGAGCAGC
AFUA_1G10060	AHPA-R	CGTTCAAATCACCGGGGAGATCAC
AFUA_1G10080	ZAFA-D1	GGCAAGTCATTTACCGACAGC
AFUA_1G10080	ZAFA-R1	TCGATGACTTGACATGTTGGACG
AFUA_1G10130	SAHA-D	GTCGTCTGGTCAACCTTGGCTG
AFUA_1G10130	SAHA-R	GTGGTGCCGAACTCGACGTACTTC
AFUA_1G12830	NIAD-D	CTGTGTCGAGCCGAACTTGATGC
AFUA_1G12830	NIAD-R	CGAACTCCTTCAGAAGTGCTTCACCG
AFUA_1G13490	SPE3-D	CCGAGAACCAATGGCTGCATCTG
AFUA_1G13490	SPE3-R	TGCAGCAGACCATGAAGCCGATC
AFUA_1G13510	FACB-D	GCTGGCCGTCTAAGACTTGTTATGC
AFUA_1G13510	FACB-R	CGACGAGAAAGGTGTCTGATCAAGAG
AFUA_1G14550	SODC-D1	GGATGGGGTTGGTTAGTGACCG
AFUA_1G14550	SODC-R	CTGAAGGTAGTAAGCATGCTCCCAC
AFUA_1G15590	SDH4-D	CCAAGACCAAGACGTTCCTGGAC
AFUA_1G15590	SDH4-R	TGATGGCCTCTGTGATACCAACATCG
AFUA_1G17190	SIDI-D	GCCATCTTCGATGAGGAGGGTTACTG
AFUA_1G17190	SIDI-R	GATTACGGAGGCTACCTCGATGGC
AFUA_2G00320	ERG3B-D	GCTATTCGACAAGCAGCAACGGC
AFUA_2G00320	ERG3B-R	GTCTTGCTCATAGCACCGATCGTCAG
AFUA_2G01260	SRBA-D1	GTGCATGTGGCCGACTATTCCTGTG
AFUA_2G01260	SRBA-R	CAAGCTTCTGGCAGTAGCGTCATCATC
AFUA_2G03010	COX4-D	TGACGGTATCTCGTGATCGTCCTATCG
AFUA_2G03010	COX4-R	GGTACCAGTACTCAGGCTTGACGTAG
AFUA_2G03700	HMG1-D	CGCTCGGCAACTTGCTCGAATTG
AFUA_2G03700	HMG1-R	GTAGAAGTCCGTGTCGTAGCAGCAC
AFUA_2G03860	ZRFB-D	ACCGGCAGAAGAAGCATTGA
AFUA_2G03860	ZRFB-R	ACCGCATCACCATCAACTCA
AFUA_2G07680	SIDA-D1	ACTCCTGAGCAAGGTTCAACACTTGAG
AFUA_2G07680	SIDA-R1	GCCAGTACCGACAGCAAACTGTCAC
AFUA_2G08740	qZRFF2-D	CGTATTCCCTCTCATGTCGTCG
AFUA_2G08740	qZRFF2-R	AGAGCCATTTGCCTGGTTCG
AFUA_2G09870	TIF35-D	CGAGAGAATGGCTGGCAAGTACG
AFUA_2G09870	TIF35-R	GCGACCGAAACGCTCGAACAAG
AFUA_2G10360	BNA5-D	TGACCCTCAAACCGAGAATTCCGAG
AFUA_2G10360	BNA5-R	TCAACGATTCAAGGACACGGTCGAG
AFUA_2G11120	GTMA-D	AGTCATCCCTGTGCCGAAAGAGAC
AFUA_2G11120	GTMA-R	ATCCTTCTCGTGCGATTCTCGCTG
AFUA_2G14570	ZRCC-D	AGGATCCGAGCGTTACATGCGTC
AFUA_2G14570	ZRCC-R	GGACCCTGAGGGTTCATCATTGC
AFUA_2G15010	RIM15-D	GCCAAGTTACTCCTCCGATCATCGTG
AFUA_2G15010	RIM15-R	GGCTTCGGAGTCAGTAGTCCGATTG

Gene ID	Oligonucleotide	5'-sequence-3'
AFUA_2G15290	AFX3-D	GGAAAGGCTGTTCATCGTATGTTCTGC
AFUA_2G15290	AFX3-R	CAGAAAGGAAGCTTGCTAACGGTCCAG
AFUA_2G17550	AYG1-D	AAGCACGGGTACAAGACCGTCG
AFUA_2G17550	AYG1-R	ATGCTCAAACAGCACCAGGCAGTC
AFUA_3G02270	CAT1-D	ATGCAATCCAATTTGACGCTGTGGTC
AFUA_3G02270	CAT1-R	CCGAACCGGAATCCGTCAATGAG
AFUA_3G03640	MIRB-D1	TGCCATGCCTGACTTGAGCTTGATC
AFUA_3G03640	MIRB-R1	GATGAAGCCCAGAACCATGAATGCAG
AFUA_3G14440	COX9-D	CGTGGTCTTGTTCTAGACCTGAGC
AFUA_3G14440	COX9-R	GATGGAGAATCAAGTCTATGCCTGCTG
AFUA_4G03410	FHPA-D	TGGATCTGAGCCAGTTGGACAGC
AFUA_4G03410	FHPA-R	CATTCAGCATGTCCGTCATGAACGTG
AFUA_4G03460	SRBB-D1	CTCGCTGAAGAAGTCGGAGATCCTG
AFUA_4G03460	SRBB-R1	GGTGCGAAACTTGTCGTTCTCCTTG
AFUA_4G03930	CYS4-D	ACTCGCATACTCCAAAGCCA
AFUA_4G03930	CYS4-R	TGACCAAGACATGCTGACCA
AFUA_4G06530	METR-D	GCTGGAGCTGGAGAATCAATGGC
AFUA_4G06530	METR-R	GACTCGGAACTACTCCGTTCAGCG
AFUA_4G09560	ZRFC-D	CAAACTCTCGGTGCTCGTCA
AFUA_4G09560	ZRFC-R	GAAGACAATCACCACCAGCA
AFUA_4G09580	ASPF2-D1	ACCTGATGCACCGTCTGTACCATG
AFUA_4G09580	ASPF2-R1	CGAAATACTGCAACGCCTCCGAGTC
AFUA_4G10730	RVB1-D	ACAGGGTGGACAGGACGTCATGAG
AFUA_4G10730	RVB1-R	AGAGCACGCCAGGTACAAGTTCAG
AFUA_4G12840	MDE1-D	TGCATTCGAGGAGGATCTCACCG
AFUA_4G12840	MDE1-R	CTGTGTCTTGGCTTTGGCAACGTC
AFUA_5G01030	GPDB-D1	GGATCGGTCGCATTGTCTTTCGC
AFUA_5G01030	GPDB-R1	GAGCCTTCGTCCTGGGAGATTTCG
AFUA_5G01970	GPDA-D	GGCGAGCTCAAAAACATCCTCGG
AFUA_5G01970	GPDA-R	CACTCGTTGTCGTACCAGGCG
AFUA_5G02180	CYSB-D	CCATCAGCGACGAAAAGTCCATTGAG
AFUA_5G02180	CYSB-R	CATACCGGTAAGCACCATCGCAC
AFUA_5G03800	FTRA-D	CGTCTGGCATGTCAACTGCTGTAG
AFUA_5G03800	FTRA-R	TGACGCATAGCCACAAACCAGACG
AFUA_5G03920	HAPX4-D	TGCGGCGTTCAAGGAACAGATCAC
AFUA_5G03920	HAPX3-R	AGCCTCCCTGTCCGAAAGAGACG
AFUA_5G04130	PHOA-D	TCGAATGCTCCAATTACGGCCAG
AFUA_5G04130	PHOA-R	TGTTGTTGCTGCAACTGAGCCTG
AFUA_5G06240	ALCC-D	AGGACCTTCCGCGTATATTTGAGCTG
AFUA_5G06240	ALCC-R	GTCAATCACCAATCGCTGCTATCTGC
AFUA_5G06270	HEMA-D	CTGGAGGAGCACGGAATCTACGTC
AFUA_5G06270	HEMA-R	GCGCTTGATACCCAGATCGTTCCAG
AFUA_5G08090	PYROA-D	GTGAGCTTGAGGTCCCTTATGAGCTTC
AFUA_5G08090	PYROA-R	CGCTTCTTCGCATCACCAGACTTG
AFUA_5G09240	SODA-D2	TTAAGCTGATTGGTGCCGAGAGCG
AFUA_5G09240	SODA-R2	GCTTAAGCGGCGATACCAATGACAC
AFUA_5G09360	CALA-D	GGGAGGAGTCTGAGAGAGTTACGG
AFUA_5G09360	CALA-R	TGGAAGACGCTCGTTCTGCAAGTC
AFUA_5G09680	SDH3-D	TCCCATTGCTGCCAAGGTTCTCATC
AFUA_5G09680	SDH3-R	AGTCCAGCCAGACTTGATAACCTGC
AFUA_5G10560	COX5A-D	GAAGAAGGCTGCCTACTGGATTGC
AFUA_5G10560	COX5A-R	CTGCCACTCCTTGGTCATGGTC
AFUA_6G00690	AFX2-D	TACTAGCGGGTCACAGAGGGTCAC
AFUA_6G00690	AFX2-R	CATCACTGCAAACTGCGCAGAGC
AFUA_6G04430	ADI1-D	ACGAAGACGAGGAGATCCGCTAC

Gene ID	Oligonucleotide	5'-sequence-3'
AFUA_6G04430	ADI1-R	TGTCATCGGTGGTGAAACGATGGTAG
AFUA_6G04740	qACT3-D	CCACGTCACCACTTTCAACTCCATC
AFUA_6G04740	qACT3-R	TCCTTCTGCATACGGTCGGAGATAC
AFUA_6G05160	AZF1-D	GCCACTACGCTAAGAACGTTGACTCTG
AFUA_6G05160	AZF1-R	AGATCCTCCGATCCTTGCCACG
AFUA_6G07720	ACUF-D	AACGTGGAGTACGAGACCTACGATG
AFUA_6G07720	ACUF-R	TTGTTGACCTCGTCGGTGAAGC
AFUA_6G07980	CDC28-D	GCTCATTGACCGTGATGGCAATCTG
AFUA_6G07980	CDC28-R	CAGTAGAGTACTGACGACCACCAAGC
AFUA_6G09630	GLIZ-D1	GTCCCGAAATCCACCAACTCTG
AFUA_6G09630	GLIZ-R	AGAGGTGAGAACAGCCTACAGC
AFUA_6G09710	GLIA-D	GCCTTCACCAACAAGCTGGTCC
AFUA_6G09710	GLIA-R	CCAGCCACATACGCCTCGAGAATC
AFUA_6G09740	GLIT-D	TCGCCAAGACCGTCATTGTTCATC
AFUA_6G09740	GLIT-R	TTCTGTCATATTCAAGGCGAGCTGC
AFUA_6G09930	YAP1-D	GCGAGGACAAGTCGCAAATGCTC
AFUA_6G09930	YAP1-R	TCTGGCTTTGGTGCGCAATTCTG
AFUA_7G00250	TUBB2-D	TCAGTATCCATACCAAGAACTCCG
AFUA_7G00250	TUBB2-R	AACATGGCCGTGAACTGGGTCG
AFUA_7G02560	DLD1-D	CGAGGCAACGAAGCAGGATATGACAG
AFUA_7G02560	DLD1-R	CAACTGCTCGCTTGACCATTCGG
AFUA_7G04730	ENB1-D	GACCTCAAGTCCAATGCCACGTC
AFUA_7G04730	ENB1-R	GCAATGGCAGGGATTCCCAGAATCAG
AFUA_7G05070	FRDA-D	TGACGCCGGTCATTCATTTCACG
AFUA_7G05070	FRDA-R	ACACCACACACTCCAGAAGAGACG
AFUA_7G06570	qZRCA-D	TGCAGAGTGTTCCTCTCGGAGTCG
AFUA_7G06570	qZRCA-R	TCGCCAGATATGCAGTTCATGGACG
AFUA_7G06790	YCT1-D	ACACCTCGCAACAACTTCCT
AFUA_7G06790	YCT1-R	ATGGCAGCGGAAACAAGGAT
AFUA_8G02450	GZBA-D	CGACTTCTGATGACCACGACCACG
AFUA_8G02450	GZBA-R	CCAATGGTCGTTGTGCGGTTTGC
AFUA_8G02620	qMCHC-D	CATGCTAACGATGGGATGCG
AFUA_8G02620	qMCHC-R	CTTCGGTCTCCCAATGGTGG
GFP	qGFP2-D	CATGCCCGAAGGCTACGTC
GFP	qGFP2-R	CCGTCCTCCTTGAAGTCGATG
LUC	qLUC-D	ATGATCATGTCCGGCTACGTC
LUC	qLUC-R	CCCTTGTACTTGATCAGGGACTTC
