# Supplementary material for: The Transcription Factor ZafA Regulates the Homeostatic and Adaptive Response to Zinc Starvation in Aspergillus fumigatus
Source: Genes (Basel). 2018 Jun 26;9(7):318. doi: 10.3390/genes9070318 (PMC6070888; doi:10.3390/genes9070318)
Supplement: Supplementary file 1 [file genes-09-00318-s001.zip › Supplementary files/Table S2.pdf]

Table S2. Oligonucleotides used in this study for construction of plasmids and validation of fungal strains.

Oligonucleotide	5'-sequence-3'
JA007	CATAGCTTTCGGAATCATCCGACTCG
JA181	CGGGAATTCGATTAGTGCTAGCAAAAACTTCAAATGTTCGG
JA182	CGGGAATTCGATTCCAACTCCAATGTC
JA194	GGCGCCGTTAACCCATTTGTGAGTACAT
JA196	TCTAGAAGATCTGGCCATGATTGCTTGG
JA197	TGTCTCCATCCAGATTGGCACGC
JA204	TAGGATTGACCGCCTGCAGTGTGC
JA261	TGGTACCTCTAGATGGCCATGGTGAGGCGAGGTGATAGG
JA274	ATAAAGACCCCCTCAGTTCTCAGTCTCAC
JA275	GTGAGACTGAGAACTGAGGGGGTCTTTAT
JA298	AGATCCGCCACAACATCGAGGACGGC
JA375	TGTACACGCGCCCATATGCAAG
JA376	TGTACACAATGCATGGAGGC
JA377	CTTTCGCCGTGAACACACCAC
JA378	ACAGGCTAACGCTTGTGATGAC
JA379	GACATGGTTGCATCATCCGCATCG
JA380	TCGGACATAACGAACGACTC
JA381	ACTCCCTTGGATCAAGCTGTGG
JA388	ATGTCCGCACGGAGAGCCACAAAC
JA394	GCCTGACAATTCAGGCGATACG
JA439	6FAM-GAGGGAAAGGGGCCCTGGTAATAC
JA463	GAAGTCCCTGATCAAGTACAAGGGCTAC
JA505	GCCGTTAGGGATATCTGCAATGAGTGCGT
FAM-Fw	6FAM-ACGACTCACTATAGGGCGAATTGGG
FAM-Rv	6FAM-CAGCTATGACCATGATTACGCCAAGC
Fw	ACGACTCACTATAGGGCGAATTGGG
Rv	CAGCTATGACCATGATTACGCCAAGC
