# Supplementary material for: The Transcription Factor ZafA Regulates the Homeostatic and Adaptive Response to Zinc Starvation in Aspergillus fumigatus
Source: Genes (Basel). 2018 Jun 26;9(7):318. doi: 10.3390/genes9070318 (PMC6070888; doi:10.3390/genes9070318)
Supplement: Supplementary file 1 [file genes-09-00318-s001.zip › Supplementary files/Table S7.pdf]

Table S7. Control genes used for MEME analysis

Gene ID	Type of gene
AFUA_1G02580	SSA
AFUA_1G04190	SSA
AFUA_1G04930	Non influenced by zinc starvation
AFUA_1G04950	Non influenced by zinc starvation
AFUA_1G06060	Non influenced by zinc starvation
AFUA_1G06860	SSA
AFUA_1G07160	Non influenced by zinc starvation
AFUA_1G08790	Non influenced by zinc starvation
AFUA_1G09800	SSA
AFUA_1G10880	SSA
AFUA_1G11930	SSA
AFUA_1G12000	SSA
AFUA_1G13570	SSA
AFUA_1G13610	SSA
AFUA_1G13620	SSA
AFUA_1G14730	Non influenced by zinc starvation
AFUA_1G16780	Non influenced by zinc starvation
AFUA_2G01550	Non influenced by zinc starvation
AFUA_2G02730	Non influenced by zinc starvation
AFUA_2G03140	SSA
AFUA_2G03390	Non influenced by zinc starvation
AFUA_2G04650	Non influenced by zinc starvation
AFUA_2G08850	Non influenced by zinc starvation
AFUA_2G10270	SSA
AFUA_2G10340	Non influenced by zinc starvation
AFUA_2G10730	Non influenced by zinc starvation
AFUA_2G12030	SSA
AFUA_2G13240	SSA
AFUA_2G13490	Non influenced by zinc starvation
AFUA_3G03620	Non influenced by zinc starvation
AFUA_3G04150	Non influenced by zinc starvation
AFUA_3G04160	Non influenced by zinc starvation
AFUA_3G06970	SSA
AFUA_3G07590	SSA
AFUA_3G07940	SSA
AFUA_3G08010	Non influenced by zinc starvation
AFUA_3G10310	SSA
AFUA_4G05940	SSA
AFUA_4G06870	SSA
AFUA_4G07420	SSA
AFUA_4G08730	SSA
AFUA_4G10780	Non influenced by zinc starvation
AFUA_4G12080	SSA
AFUA_4G12830	Non influenced by zinc starvation
AFUA_5G02360	Non influenced by zinc starvation
AFUA_5G04240	SSA
AFUA_5G05740	SSA
AFUA_5G05790	Non influenced by zinc starvation
AFUA_5G05920	SSA
AFUA_5G07050	Non influenced by zinc starvation
AFUA_5G07100	Non influenced by zinc starvation
AFUA_5G08330	SSA
AFUA_5G11240	Non influenced by zinc starvation
AFUA_5G11820	Non influenced by zinc starvation

Gene ID	Type of gene
AFUA_5G12130	SSA
AFUA_5G13920	SSA
AFUA_6G01930	SSA
AFUA_6G04290	SSA
AFUA_6G04600	Non influenced by zinc starvation
AFUA_6G04680	SSA
AFUA_6G06860	Non influenced by zinc starvation
AFUA_6G07640	Non influenced by zinc starvation
AFUA_6G10460	SSA
AFUA_6G12370	Non influenced by zinc starvation
AFUA_6G13945	SSA
AFUA_7G01860	SSA
AFUA_7G05260	SSA
AFUA_7G05930	Non influenced by zinc starvation
AFUA_8G00100	SSA
AFUA_8G02780	Non influenced by zinc starvation
AFUA_8G04270	SSA
