# Supplementary material for: The Transcription Factor ZafA Regulates the Homeostatic and Adaptive Response to Zinc Starvation in Aspergillus fumigatus
Source: Genes (Basel). 2018 Jun 26;9(7):318. doi: 10.3390/genes9070318 (PMC6070888; doi:10.3390/genes9070318)
Supplement: Supplementary file 1 [file genes-09-00318-s001.zip › Supplementary files/Table S8.pdf]

Table S8. All possible ZR motifs harboring one substitution out of the conserved hexanucleotide core (5'-CARGGT-3') with respect to the ZR consensus motif (ZR0).

ZR	DNA sequence
ZR0	DYYVYCARGGTVYYY
ZR1	CYYVYCARGGTVYYY
ZR2	DRYVYCARGGTVYYY
ZR3	DYRVYCARGGTVYYY
ZR4	DYYTYCARGGTVYYY
ZR5	DYYVRCARGGTVYYY
ZR6	DYYVYCARGGTTYYY
ZR7	DYYVYCARGGTVRYY
ZR8	DYYVYCARGGTVYRY
ZR9	DYYVYCARGGTVYYR

D = G or A or T
V = G or A or
R = G or A
Y = C or T
